# A urinary extracellular vesicle microRNA biomarker discovery pipeline; from automated extracellular vesicle enrichment by acoustic trapping to microRNA sequencing

**DOI:** 10.1371/journal.pone.0217507

**Published:** 2019-05-29

**Authors:** Anson Ku, Naveen Ravi, Minjun Yang, Mikael Evander, Thomas Laurell, Hans Lilja, Yvonne Ceder

**Affiliations:** 1 Department of Translational Medicine, Lund University, Malmö, Sweden; 2 Division of Clinical Genetics, Department of Laboratory Medicine, Lund University, Lund, Sweden; 3 Department of Biomedical Engineering, Lund University, Lund, Sweden; 4 Urology Service, Department of Surgery, Memorial Sloan Kettering Cancer Center, New York, New York, United States of America; 5 Genitourinary Oncology Service, Department of Medicine, Memorial Sloan Kettering Cancer Center, New York, New York, United States of America; 6 Department of Laboratory Medicine, Memorial Sloan Kettering Cancer Center, New York, New York, United States of America; 7 Nuffield Department of Surgical Sciences, University of Oxford, Oxford, United Kingdom; 8 Division of Translational Cancer Research, Department of Laboratory Medicine, Lund University, Lund, Sweden; Institut de Pharmacologie Moleculaire et Cellulaire, FRANCE

## Abstract

Development of a robust automated platform for enrichment of extracellular vesicles from low sample volume that matches the needs for next-generation sequencing could remove major hurdles for genomic biomarker discovery. Here, we document a protocol for urinary EVs enrichment by utilizing an automated microfluidic system, termed acoustic trap, followed by next-generation sequencing of microRNAs (miRNAs) for biomarker discovery. Specifically, we compared the sequencing output from two small RNA library preparations, NEXTFlex and CATS, using only 130 pg of input total RNA. The samples prepared using NEXTflex was found to contain larger number of unique miRNAs that was the predominant RNA species whereas rRNA was the dominant RNA species in CATS prepared samples. A strong correlation was found between the miRNA expressions of the acoustic trap technical replicate in the NEXTFlex prepared samples, as well as between the acoustic trap and ultracentrifugation enrichment methods. Together, these results demonstrate a robust and automated strategy for biomarker discovery from small volumes of urine.

## Introduction

Extracellular vesicles (EVs) are secreted cellular products that harbor proteins, lipids, DNA and RNA, especially small RNA such as microRNA (miRNA) within the phospholipid bilayer that protects them from the degradative effect of circulating proteases and nucleases [1]. Recent research has shown that miRNAs in EVs play a pivotal role in the progression of various diseases. In cancer, intravesicular miRNAs have been shown to confer drug resistance to surrounding tumor cell population, induce local vascular expansion, trigger immune-shielding and prime distant metastatic niches [2]. As a result of its systemic availability and biological role, EV miRNAs could serve as a valuable source of biomarkers and provide prognostic information.

**Fig 1:**
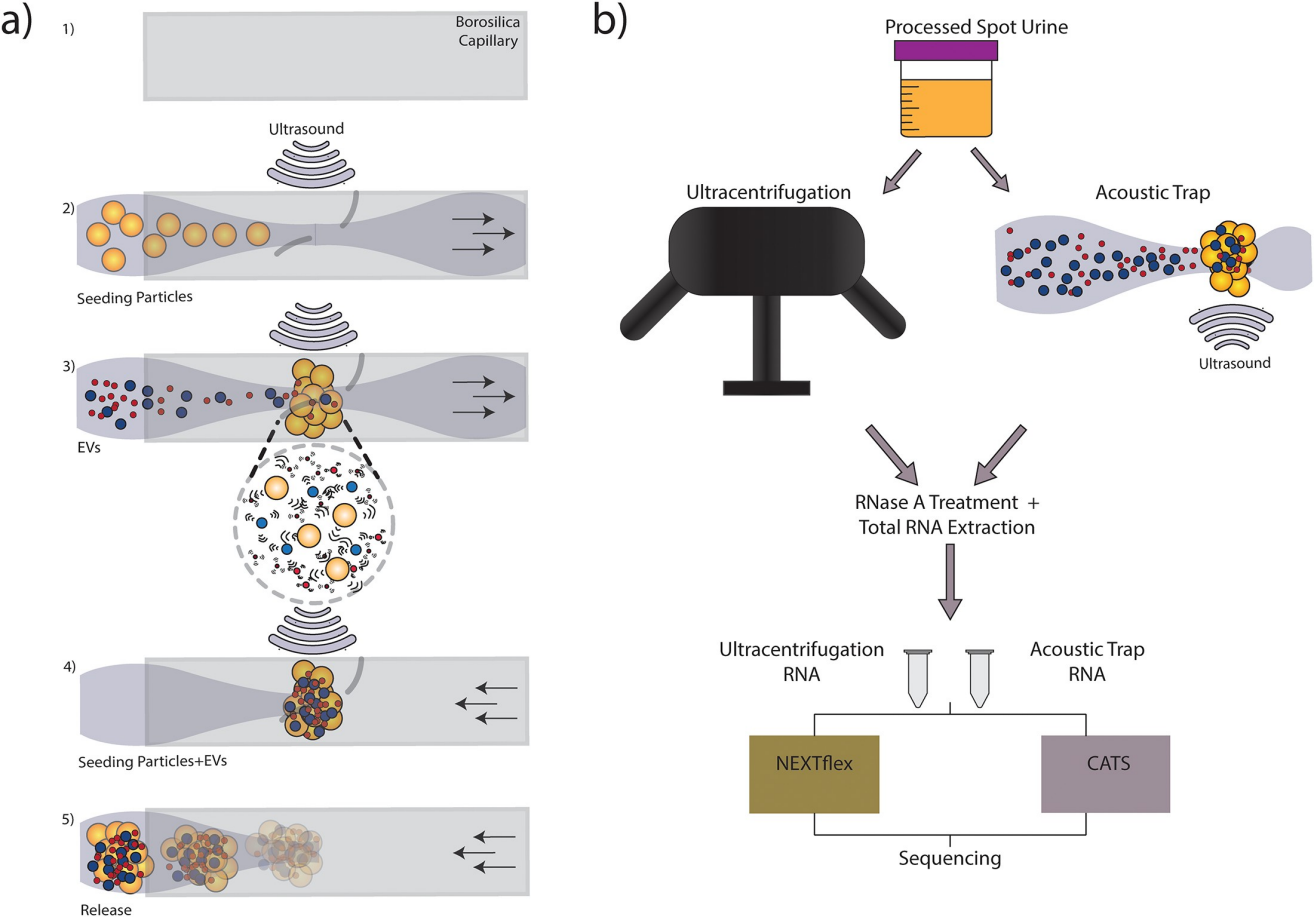
Illustration of acoustic trapping procedure and experimental workflow. a) The acoustic trapping was performed in a 1) boroscilicate glass capillary, 2) ultransonic wave (dashed grey line) is generated by an ultrasonic transducer while seeding particles (yellow spheres) are aspirated (black arrow) and trapped in the glass capillary, 3) the EVs in the samples are captured by secondary acoustic forces reflected off seeding particles (magnified view below), 4) washing of the EVs and seeding clusters, and 5) release of EVs and seeding particle. b) Healthy human urine was collected and processed by centrifugation. Samples were aliquoted for EVs enrichment by either ultracentrifugation (UC) or acoustic trap (AcT). Total RNA was extracted after RNase A treatment followed by library preparation with either NEXTflex or CATS preparation kit. The samples were sequenced using Illumina’s Nextseq platform.

At present, the established EV enrichment method is differential centrifugation [3]. Unfortunately, implementation of differential centrifugation for EV enrichment in clinical practice has been restricted, in part due to the volume requirements, labor-intensive work up and low reproducibility [4]. Therefore, there is an important need to develop an EV enrichment method that is reproducible, rapid, less labor-intense, and require small sample volume in order to facilitate the translation of research findings into the clinic. Recently, we have developed acoustic trapping, an automated, non-contact EV enrichment microfluidic technology that utilizes the scattering of ultrasound waves off large pre-seeded polystyrene particles to capture EVs (Fig 1a) [5–7]. In acoustic trapping, samples are passed through a borosilicate glass capillary to the ultrasonic resonant region where the presence of large pre-seeded particles leads to the scattering of acoustic waves that draws the EVs and the pre-seeded particles together. The phenomenon is dependent on inter -particle distance, density and compressibility of the EVs. A pilot study using the acoustic trap technology was recently reported for urine, plasma and conditioned media where we confirmed the presence of EV surface markers such as CD9/63/81 and intravesicular miRNAs [8].

Currently, next-generation sequencing is the most widely used method for miRNA discovery due to the high sensitivity and ability to detect different isoforms. There are challenges associated with miRNA sequencing, mainly during library construction owing to the short length, secondary structure, and high GC content of miRNA [9–12]. In addition, miRNA’s nucleotide composition at the 5’- and 3’-ends have been shown to reduce the ligation efficiency that leads to dropouts during library preparation [13]. To overcome that hurdle, commercial miRNA sequencing kits employ strategies such as random nucleotide sequences within the adapter sequence or by polyadenylation of the 3’-end [14]. However, there is still a gap of knowledge regarding the use of miRNA library construction methods toward minute quantities RNA that are commonly obtained during EV research.

Here we investigated a comprehensive biomarker discovery pipeline for miRNA derived from both small and large urinary EVs. We utilized the acoustic trap instrument to enrich urinary EVs and then compared the libraries obtained from two commercially available, low-input compatible, small RNA library kits, followed by next generation sequencing. In order to assess if the acoustic trap could replace ultracentrifugation as the preferred EV-enrichment method, we investigated the reproducibility of acoustic trapping and compared the miRNAs content of the EVs obtained using the different enrichment methods.

## Materials and methods

### Sample collection and processing

A single spot urine sample collected from a heathy male donor with written consent and approval by Regional Ethical Review Board in Lund # 2013–400 was immediately centrifuged at 2,000xg for ten minutes at room temperature to remove cell debris while preserving the small and large EVs in the sample. A total of 72 mL of supernatant was aliquoted for EVs enrichment by ultracentrifugation; spun twice at 100,000xg for 1.5 h at 4°C with a PBS wash in-between centrifugation using a SW41-Ti swing bucket rotor in an Optima XE-90 (Beckman Coulter, USA). The pellets were resuspended in 330 μL PBS and stored at -80°C. Acoustic trapping was performed as described previously [8], briefly, ultrasonic transducer was set to operate at 10 V_pp_ and 4.2 MHz. Next, 12 μm seeding particles were trapped and washed with PBS follow by aspiration of 0.75 mL of urine sample at 15 μLmin^-1^ into the capillary for EV enrichment then washed with 200 μL PBS prior to elution with 30 μL PBS. A total of 9.75 mL of urine samples were trapped in eleven fractions, totaling 330 μL of purified sample volume. Nanoparticle-tracking analysis (NTA) was performed using NanoSight LM10 (Malvern, UK) on urine samples diluted eight-times (0.125x of stock) with PBS using continuous flush mode and measured five times. Camera level was set to ten and gain set to one.

### RNA extraction and quantitation

Enriched EVs were treated with 10 μg/mL of RNase A for 10 min at 37° prior to RNA extraction with Single Cell RNA Extraction kit (Norgen, Canada) per manufacturer’s plasma sample protocol RNA were eluted in a total of 30 μL of elution buffer per sample. Purified RNA was used as a positive control for RNase A activity (S1A Fig). To estimate RNA extraction efficiency, 3x10^8^ copies of cel-miR-39 synthetic spike-in (Qiagen, Denmark) was added before RNA extraction. The quantity and size profile of the extracted RNA were determined by Qubit RNA HS assay (Thermo Fisher Scientific, USA) and the Pico RNA chip of the Bioanalyzer (Agilent, USA) respectively. Quantitation of miRNA levels was performed by qRT-PCR using TaqMan assay (Applied Biosystems, California) with cel-miR-39, -16, -21, and -24 specific primers (TaqMan microRNA Assay) on QuantStudio 7K system (Life Technologies, USA). The RNA concentration of the acoustic trap was estimated by the following formula:

$${\text{C}}_{\text{AcT}}={\;\text{C}}_{\text{UC}}\;\text{x}\;\left(1/\text{n}\right)\;\text{x}\sum 2^{-{\text{dCt}}_{\text{miR}}},$$

where C_AcT_ is the estimated RNA concentration of acoustic trap sample, C_UC_ is the RNA concentration of ultracentrifugation sample determined by Qubit RNA HS kit, n is the number of miRNA excluding the spike-in and $2^{-{\text{dCt}}_{\text{miR}}}$ is the miRNA expression fold-change in acoustic trap samples compared to ultracentrifugation samples.

### Library preparation and sequencing

Three libraries were constructed using NEXTflex small RNA library kit (Perkin Elmers, USA) and the CATS small RNA library (Diagenodes, USA). The three libraries consist of two technical replicates from the acoustic trap and an ultracentrifugation sample per manufacturer’s protocol using an estimated 130 pg of input RNA for both acoustic trap replicates and ultracentrifugation. Briefly, NEXTFlex libraries were constructed by 3’- and 5’-adapters ligations followed by reverse transcription and 22 cycles of PCR with barcodes. Cleanup was performed with manufacturer provided kit. CATs libraries were constructed by dephosphorylation and polyadenylated at the 3’-end followed by cDNA strand synthesis with poly(dT) primer containing the P7 adapter sequence. P5 adapter sequence is incorporated onto the 5’-end of the RNA after template switching follow by PCR of 15 cycles. Cleanup was performed using Agencourt AMPure XP (Beckman Coulter, USA). The size, concentration and adapter-dimer fraction of the cDNA products were calculated from the electropherogram of the Bioanalzyer and Qubit RNA HS assay (Thermo Fisher Scientific, USA). Paired-end sequencing of 75 bp was performed on the NextSeq 500/550 platform (Illumina) using a mid-output flow cell with 10% PhiX spike-in.

### Data processing and statistics

Fold change from qRT-PCR were calculated using the ΔΔCt follow by log2 transformation in quadruplicates. Adapters were trimmed per manufacturer protocol. Sequences were annotated using the Unitas pipeline v1.5.2 allowing one mismatch and greater than 15 nt in length to miRbase v22, human tRNA piRBase v2.0, coding gene in ensembl Database release 92 [15, 16]. Set analysis of miRs between ultracentrifugation and acoustic trap replicates was performed by intersection of miRs with greater than zero counts. All correlation analysis was performed by non-parametric Spearman correlation. Trimmed mean method (TMM) normalization and differential expression analysis were performed using edgeR with at least five counts. A significance level of Benjamini-Hochberg adjusted (BH-adjusted) p≤0.05 was used in differential expression analysis.

## Results

### Urinary EV enrichment and RNA extraction

**Fig 2:**
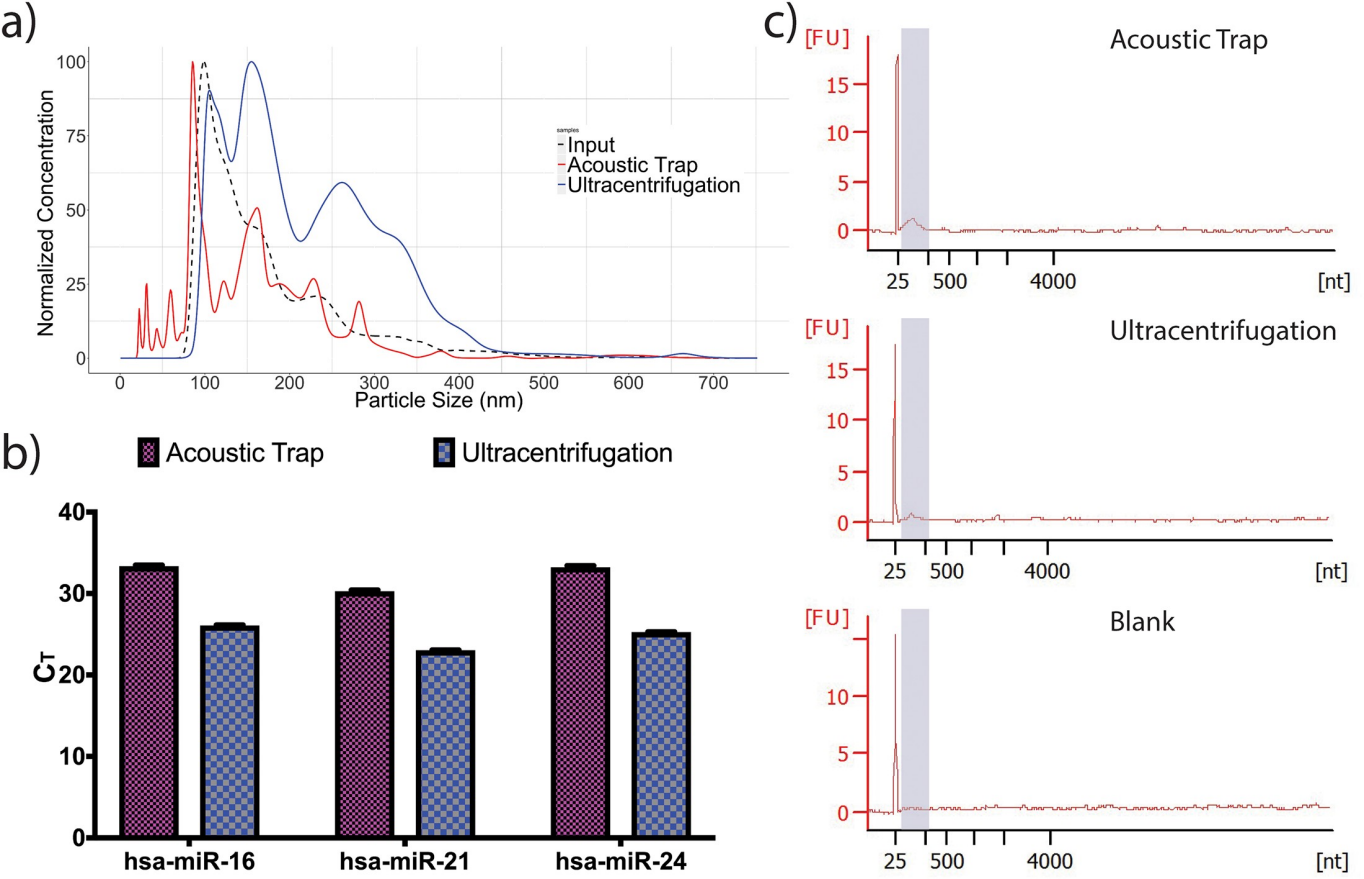
Characteristics of acoustic trap and ultracentrifugation enriched EVs and the associated RNA products after total RNA isolation. a) Representative nanoparticle tracking analysis of EVs obtained after acoustic trap (red solid line) or ultracentrifugation enrichment (blue solid line) compared to diluted input sample (dashed black line). Acoustic trap, ultracentrifugation and input EVs showed mean (±SD) Dv50 of 149 (±14), 198 (±8) and 143 (±4) nm respectively. b) Verification of intact miRNA after RNase A treatment such as hsa-miR-16/21/24 by qRT-PCR from acoustic trap and ultracentrifugation of urine prior to library construction. Note the different volume of urine aliquoted for acoustic trapping, 9.75 mL and ultracentrifugation, 72 mL. The RNA concentration of acoustic trap sample was estimated by multiplying the fold change (acoustic trap to ultracentrifugation sample) of miR-16/21/24 to the RNA concentration of ultracentrifugation sample determined by Qubit assay. c) The size profile of the isolated total RNA are predominantly small RNAs less than 500 nt and devoid of any intact rRNA or mRNA.

EVs enriched using acoustic trapping or ultracentrifugation were investigated by nanoparticle tracking analysis. Size distribution analysis showed that the mean diameter (Dv50±SD) of EVs enriched using acoustic trapping (149±14) is similar to that of the input (143±4), whereas EVs enriched by ultracentrifugation are larger (198±8). (Fig 2a). Owing to the lower detection limits of current RNA detection techniques, concentration estimates of low RNA quantities may be inaccurate. In order to address this potential pitfall, we performed EVs enrichment by ultracentrifugation based on a volume of 72 mL of urine sample to generate sufficient sample for accurate RNA detection and compared that to RNA detected in EVs enriched from 9.75 mL of urine by acoustic trap. A total of 138 ng of total RNA was isolated from the ultracentrifugation sample as determined by Qubit assay. Next, we estimated the total RNA isolated from the acoustic trapped sample by multiplying the average relative abundance of miR-16/21/24 (0.57%±0.15%) to the 138 ng of total RNA obtained from ultracentrifugation sample (Fig 2b) which resulted in 0.79 ng of total RNA. The estimation relies on the RNA size profile of the two samples to be similar, indeed, electropherogram of the RNA extracted from EVs showed that the majority of RNA are less than 200 nt in length with no discernible rRNA fractions in both samples (Fig 2c). An alternative estimate of the total RNA in the ultracentrifugation, acoustic trap sample were determined from the Bioanalyzer output which resulted in 104±61 and 90±9 pg/μL respectively and a blank sample measured at 60±30 pg/μL of total RNA (S1 Table). Adjusting for background, the total RNA of the acoustic trap sample resulted in 0.90 ng of total RNA compared to the estimated 0.79 ng. The corresponding RNA extraction efficiency is 80% for from 9.75 mL of urine acoustic trap and 56% for ultracentrifugation based on cel-miR-39 spike-in (S1B Fig). Further, the detection of hsa-miR-16/21/24 by qRT-PCR subsequent to RNase treatment confirmed the presence of intravesicular miRNA in our acoustic trapped and ultracentrifugation samples (S1C Fig and S2 Table).

### Sequencing libraries quality control

The cDNA libraries were produced from an estimated 130 pg of total RNA. Both kits produced libraries with fluorescent peaks corresponding to the miRNA fraction of ~150 nt, which is the expected size for adapters with insert (S2 Fig). Quantifying the relative molar concentration of adapters with inserts, NEXTFlex libraries produced 75%, 80% and 93% molar fraction for acoustic trap replicate 1, 2 and ultracentrifugation respectively, compared to CATs that produced 66%, 93% and 99% molar fractions respectively (S3 Table).

### Comparison of the different library preparation kits

**Fig 3:**
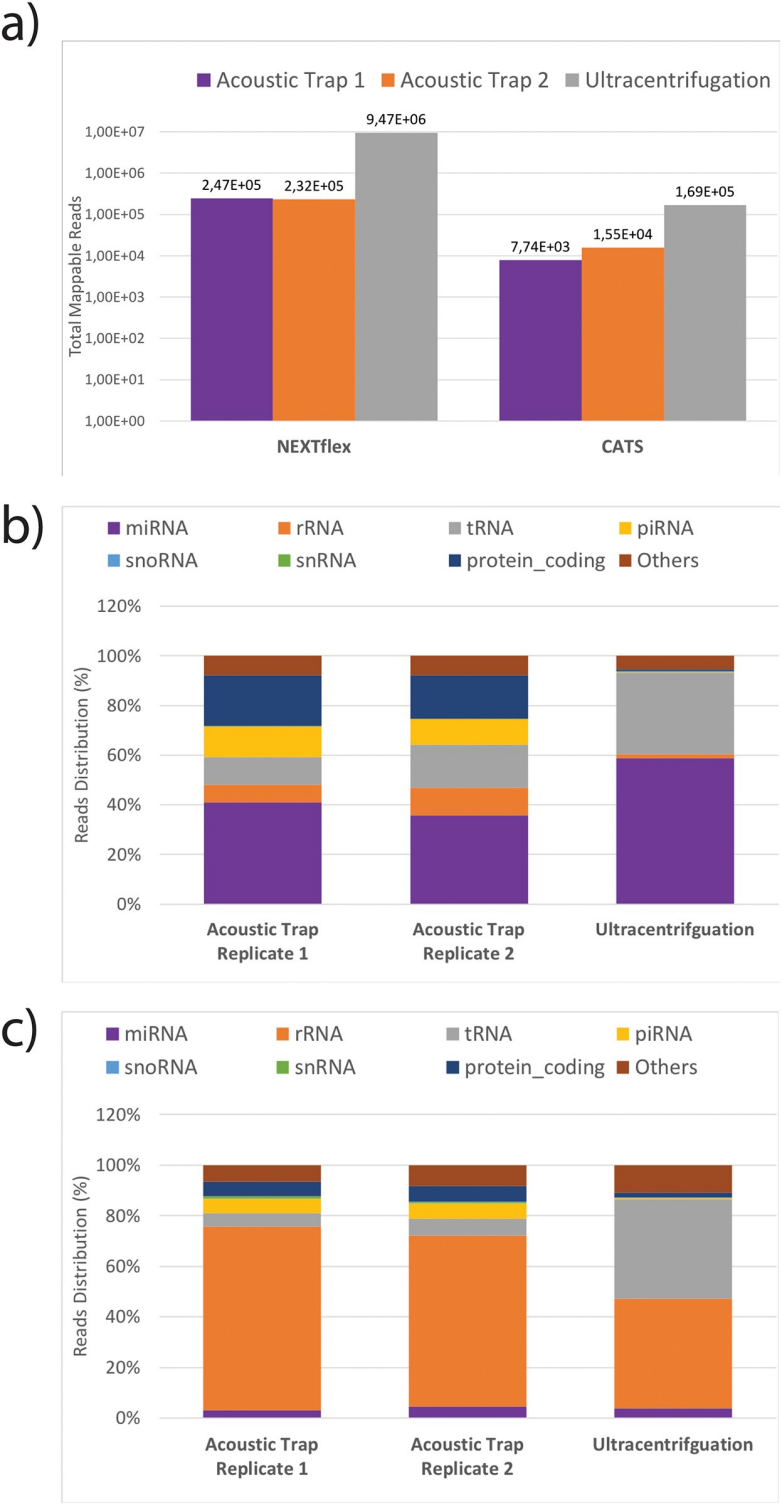
Comparison of NEXTFlex and CATS libraries reads and read composition. a) Total number of mappable reads obtained for NEXTFlex and CATS libraries. The NEXTFlex libraries yielded 2.5 x10^5^, 2.3 x10^5^ and 9.5 x10^5^ reads and the CATS libraries yielded 7.7 x10^3^, 1.6 x10^3^ 1.7 x10^5^ reads for the two acoustic trap replicates and ultracentrifugation samples respectively. b) Mapped reads from the NEXTflex libraries showing predominantly miRNA fraction. c) Mapped reads from CATS libraries are composed of mainly rRNA.

A total number of 1.1x10^6^, 1.5x10^6^ and 11x10^6^ reads were obtained for acoustic trap replicate 1, 2 and ultracentrifugation respectively for NEXTFlex libraries and 0.74x10^6^, 0.60x10^6^, 0.94x10^6^ reads for CATS libraries after adapter trimming. From the NEXTFlex libraries, 23%, 15% and 83% of the reads were mappable (Fig 3a), with miRNAs constituting the largest fraction at 41%, 36% and 59% for acoustic trap replicates 1, 2 and ultracentrifugation respectively (Fig 3b and S4 Table). Other small RNA species such as rRNA, tRNA, piRNA, snoRNA, snRNA and protein coding genes were also found at lower percentages. For the CATS libraries, only 1.3%, 1.7% and 23% of the reads were mappable with rRNA being the predominant RNA species identified at 73%, 68% and 43%, while the miRNA fractions constituted only 3%, 5% and 4% for acoustic trap replicate 1, 2 and ultracentrifugation respectively (Fig 3c and S4 Table). Investigation into the low mappable reads revealed that most of the CATS’ reads harbor cytosine or thymidine interspersing the polyadenylated sequence causing misalignment during mapping.

A comparison of the miRNAs identified in the ultracentrifugation sample of the NEXTFlex and CATS libraries showed that 114 miRNAs overlap while 393 miRNAs are exclusive to the NEXTFlex prepared samples and 28 miRNAs exclusive to CATS prepared samples (S3A Fig). The miRNA expression levels were found to correlate between the two kits using RNA extracted from EVs isolated by ultracentrifugation (S3B Fig; Rho = 0.46, p = 3.0x10^-7^). However, a similar comparison for miRNAs in EVs extracted using acoustic trap could not be performed due to the limited number of miRNAs found in CATS libraries.

### Comparison of the miRNA expression from the technical replicates

**Fig 4:**
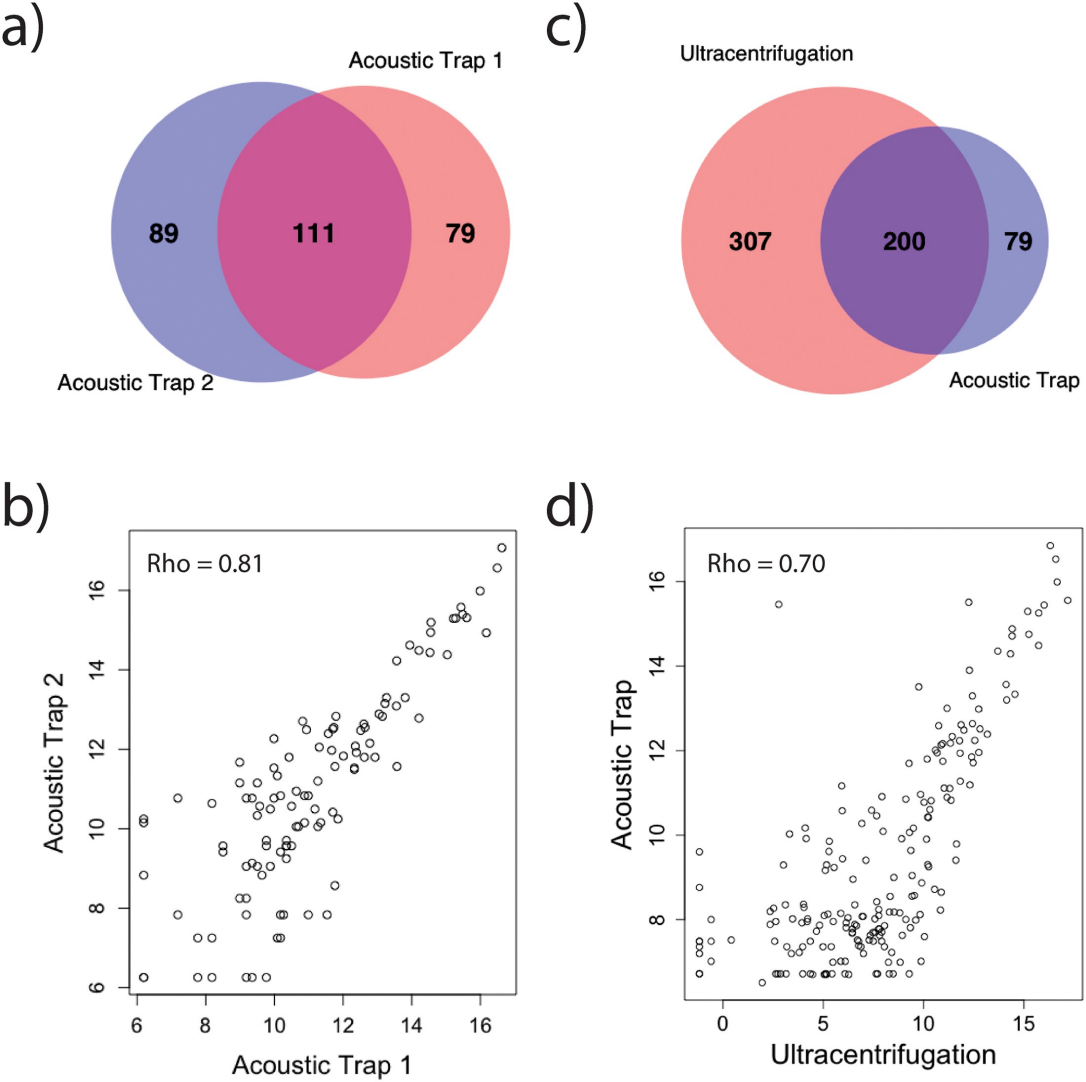
Comparison of the miRNA expression between samples. a) Venn diagram of common and exclusive miRNA found between the two replicates. Of the total of 279 miRNAs identified by the two replicates, 111 miRNAs are found in both libraries and 168 are exclusive to either of the libraries. b) Spearman correlation of the two replicates shows a significant correlation with rho = 0.81. c) Venn diagram of common and exclusive miRNA found between the acoustic trap and ultracentrifugation samples. Of the total of 586 miRNAs identified, 200 miRNAs are found in both libraries and 307 79 and 307 are found exclusively in the acoustic trap and ultracentrifugation samples respectively. d) Spearman correlation of the normalized miRNA expression of ultracentrifugation and median normalized expression of the acoustic trap replicates shows a significant correlation with rho = 0.70.

To ascertain the technical repeatability of the NEXTFlex libraries, the concordance of the miRNA expression between acoustic trap replicates was compared. A total of 190 and 200 unique miRNAs were identified from the two acoustic trap replicates. Of those miRNAs, 111 are common between the replicates while 89 and 79 miRNAs are exclusive to each replicate (Fig 4a and S5 Table). Analysis of the exclusive sets of miRNAs from each acoustic trap replicate shows that they predominantly constitute lower abundance reads after normalization (S4 Fig). Further, the concordance of miRNA expression (log normalized) of the common miRNAs was also explored by Spearman correlation and showed a highly significant correlation with rho of 0.81, p<2.2x10^-16^ (Fig 4b).

### Comparison of acoustic trap and ultracentrifugation miRNA expression

As the current standard method for EVs enrichment is ultracentrifugation, we compared the expression of 279 unique miRNAs from the acoustic trap replicates against the expression of 507 miRNAs from ultracentrifugation. Of those miRNAs, 200 are common between the acoustic trap and ultracentrifugation, while 79 miRNAs are unique to the acoustic trap and 307 miRNAs are exclusive to ultracentrifugation (Fig 4c and S5 and S6 Tables). Analysis of the miRNA expression (log normalized) of the common miRNAs showed a significant correlation between the two enrichment methods with rho of 0.70, p<2.2x10^-16^ (Fig 4d).

### Urinary EVs miRNA expression validation

To validate the miRNAs expression, we compared our data to previously reported miRNA profiles of EVs from nine healthy male donors enriched by ultracentrifugation [17] revealed that majority of the miRNAs are overlapping and the median counts correlates with Rho of 0.64, p<2.2x10^-16^ and 0.73, p< p<2.2x10^-16^ in EVs extracted by acoustic trap or ultracentrifugation, respectively (S5 Fig and S7 Table).

## Discussion

Overcoming current limitations to enrich EVs is critical for translating EV-based diagnostics into clinical practice. We previously showed that EVs, ranging widely in size from exosomes to microvesicles, can be enriched using the acoustic trap method [8]. However, prerequisites for biomarker discovery and clinical applications, such as detailed protocols incorporating an efficient and robust EV enrichment method combined with sensitive downstream sequencing pipeline are lacking. In addition, isolation of EVs from urine is particularly challenging due to the presence of Tamm-Horsfall protein that can sequester EVs and the large concentration range that EVs can present in urine samples [18]. Here we demonstrate that urinary EVs can be enriched using acoustic trapping technology and subsequently analyzed by next generation sequencing using an estimated 130–150 pg of total RNA based on Qubit and qPCR or Bioanalyzer alone.

We observed that NEXTFlex preparation resulted in more mappable reads compared to CATS, and the difference in mappable fractions between the two libraries is attributable to the presence of extraneous nucleotides in the polyadenylated flanking sequences that created a challenge for its removal using the Cutadapt tool. It is plausible that the reverse transcriptase fidelity is responsible for the observation. Others have concluded that NEXTFlex outperforms CATS [14], but to the best of our knowledge there are no previous reports showing differences in extraneous nucleotides in the polyadenylated sequences.

A central question regarding the choice of library preparation is the library variability when low quantities of RNA are used. Analysis of the acoustic trap technical replicates prepared using NEXTFlex kit demonstrated highly similar characteristics such as library adapter-dimer fractions, total number of reads and reads composition that included noncoding RNA species commonly found in urinary EVs [19]. The large number of identified miRNA exclusive to each replicate is likely a result of the sequencing depth of the two libraries and the probabilistic nature of sequencing. This hypothesis is supported by the fact that most of the miRNA exclusive to each replicate have lower counts compared to the overall miRNA expression of the sample. Thus, increasing sequencing depth by reducing unligated adapters and adapter-dimers or increasing the number of replicates in each sample could theoretically improve the number of common miRNAs and, therefore, the concordance of the two replicates.

Ultracentrifugation is used as the current standard for EVs enrichment, despite the well-known limitations to routine use in clinic practice contributed by the lack of robustness and efficiency. In our study, we noted that the ultracentrifugation sample contained higher percentage of miRNA reads. This could be due to higher enrichment of non-vesicular miRNA such as lipoproteins-bound miRNA presented in urine by ultracentrifugation as reported previously [20, 21]. In addition, more unique miRNAs were identified in the ultracentrifuged samples compared to the acoustic trap replicates. The result is likely due to the seven-fold larger starting volume and ~10-fold higher read counts from the ultracentrifugation samples compared to acoustic trap. It is known that increasing sequencing depth will significantly increase the number of detected miRNAs and the concordance of the two samples independent of the acoustic trap [22, 23]. However, the difference in read counts could also be attributed to errors in cDNA quantitation and larger adapter-dimer fractions in the acoustic trap samples (~23% compared to 7% in ultracentrifugation) which could be corrected by future use of additional gel-based size-selection. In addition, we note that based on the fold-change of miR-16/21/24, the expected miRNA yield is 4.2% of ultracentrifugation yield after adjusting for input volume differences. In that regard, we expect the efficiency will improve through on-going efforts in the design and operation of the acoustic trap such as increasing to higher harmonic frequencies of the acoustic trapping resonator and/or capillary dimension to enhance the performance of the system. Lastly, a significant portion of the miRNA identified in our study overlapped with the urinary EV miRNA profile published by Rodriguez *et al*, thus, offering additional evidence that acoustic trapping can enrich miRNA-containing urinary-EVs.

## Conclusion

This study demonstrated that by utilizing the NEXTflex library preparation kit as a downstream pipeline after automated acoustic trapping, small RNA libraries can be successfully constructed from as little as 130 pg of total RNA, equivalent to 1.7 mL of urine. Though the library preparation may benefit from additional size-selection steps to remove adapter dimers, we obtained a sufficient number of reads for acoustic trapped samples. Thus, acoustic trap, in conjunction with NEXTFlex can be used for high throughput and automated miRNA biomarker discovery or clinical workflow in the future.

## Supporting information

S1 Fig RNase treatment control and RNA extraction efficiency. a) Pure RNA after RNase A treatment at 37° for 10min shows complete degradation. b) RNA extraction efficiency of acoustic trap and ultracentrifugation determined by spike-in of cel-miR-39 prior to RNA isolation. c) miRNA are detectable from aoucstic trap and ultracentrifugation samples after RNase A treatment.
(PDF)

S2 Fig cDNA quantitation after library preparation. a) NEXTflex prepared libraries with acoustic trap replicates on the left and ultracentrifugation samples on the right. The libraries showed maximal peak (highlighted) at the expected miRNA size of ~150bp (insert + adapters) with calculated adapter-dimer (AD) fractions displayed. b) CATS prepared libraries showed maximal peak (highlighted) at the expected miRNA size of ~154bp (insert + adapters) and AD fractions displayed.
(PDF)

S3 Fig Comparison of the NEXTFlex and CATS ultracentrifugation samples. a) Venn diagram of the miRNAs identified in the ultracentrifugation samples showed 114 common and 421 exclusive miRNAs between the NEXTFlex and CATS preparations. b) Spearman correlation analysis of the ultracentrifugation miRNA expression derived from NEXTFlex and CATS preparations showed significant correlation, rho = 0.46.
(PDF)

S4 Fig Exclusive miRNAs in each of the acoustic trap replicates are attributable to lower read counts, replicate 1 (a) and replicate 2 (b). (PDF)

S5 Fig Comparison of miRNA expression between published urinary EVs and results from acoustic trap and ultracentrifugation. a) Analysis of common and exclusive miRNAs found in the acoustic trap, ultracentrifugation and Rodriguez et al. dataset b) Spearman correlation resulted in Rho of 0.64 and 0.72 respectively for acoustic trap, left and ultracentrifugation, right.
(PDF)

S1 Table RNA concentration of ultracentrifugation, acoustic trap and blank measured by Bioanalyzer RNA Pico. (PDF)

S2 Table Ct values of four miRNAs, hsa-miR-16, 21, 24 and cel-miR-39 from qRT-PCR measurements from acoustic trapped, ultracentrifugation enriched samples and spike-in. (PDF)

S3 Table Library size profile from NEXTflex and CATS prepared samples showing the estimated primer-dimer (PD)fraction of based on manufacturer’s specification. (PDF)

S4 Table Read count of different RNA species from acoustic trap and ultracentrifugation samples prepared with NEXTFlex or CATS library preparation kit. (PDF)

S5 Table Set of common and exclusive miRNAs in ultracentrifugation and acoustic trap samples with counts greater than zero, ∩ represents common sets and—represents exclusive set. Yellow hightlighted cells represents miRNA found in AcT samples but not UC that were validated and blue highlights are differentially enriched miRNA in AcT samples used for validation.
(PDF)

S6 Table Compiled miRNA raw counts from NEXTFlex libraries in descent order of expression. (PDF)

S7 Table Compiled miRNA counts of Rodriguez et al miRNA dataset from nine healthy male donors. (PDF)
