# Correction: A urinary extracellular vesicle microRNA biomarker discovery pipeline; from automated extracellular vesicle enrichment by acoustic trapping to microRNA sequencing

**DOI:** 10.1371/journal.pone.0224604

**Published:** 2019-10-24

**Authors:** Anson Ku, Naveen Ravi, Minjun Yang, Mikael Evander, Thomas Laurell, Hans Lilja, Yvonne Ceder

The following information is missing from the Funding statement: This study was also supported by the Swedish Foundation for Strategic Research (SSF) Grant Number SBE13-0049 and the Swedish Research Council (Grants: 2014–03413, 2018–03672).
