# Supplementary figures and images for: A urinary extracellular vesicle microRNA biomarker discovery pipeline; from automated extracellular vesicle enrichment by acoustic trapping to microRNA sequencing
Source: PLoS One. 2019 May 29;14(5):e0217507. doi: 10.1371/journal.pone.0217507 (PMC6541292; doi:10.1371/journal.pone.0217507)

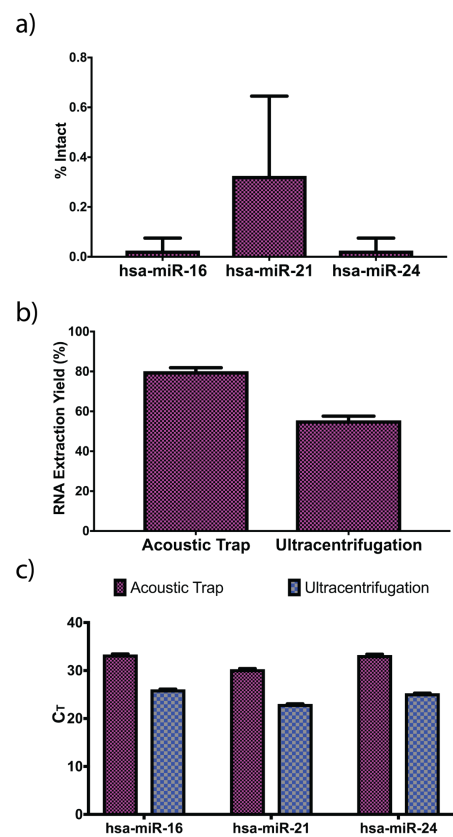

S1. Fig

Supplement: S1 Fig — a) Pure RNA after RNase A treatment at 37° for 10min shows complete degradation. b) RNA extraction efficiency of acoustic trap and ultracentrifugation determined by spike-in of cel-miR-39 prior to RNA isolation. c) miRNA are detectable from aoucstic trap and ultracentrifugation samples after RNase A treatment. (PDF) [file pone.0217507.s001.pdf]

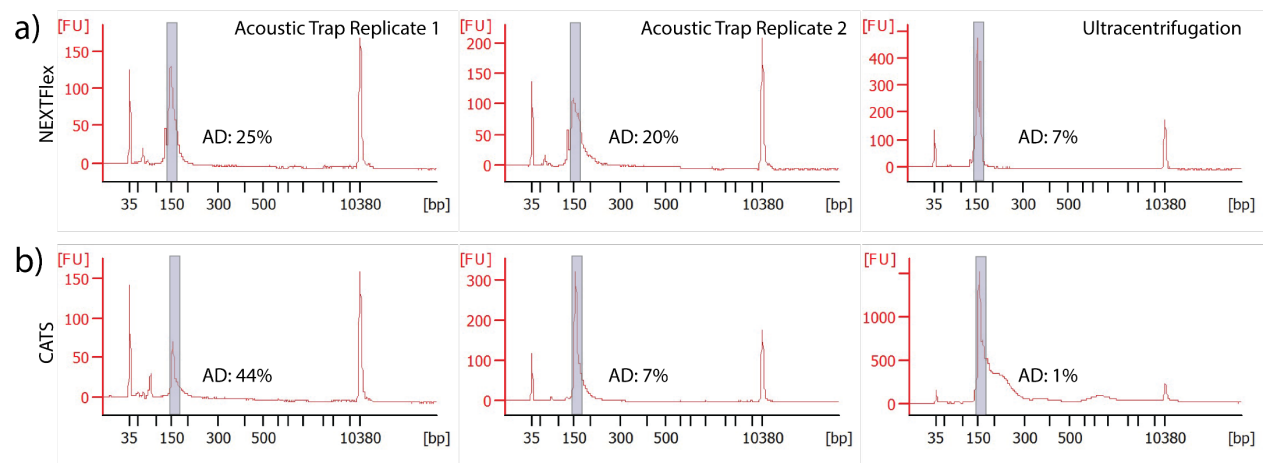

S2. Fig

Supplement: S2 Fig — a) NEXTflex prepared libraries with acoustic trap replicates on the left and ultracentrifugation samples on the right. The libraries showed maximal peak (highlighted) at the expected miRNA size of ~150bp (insert + adapters) with calculated adapter-dimer (AD) fractions displayed. b) CATS prepared libraries showed maximal peak (highlighted) at the expected miRNA size of ~154bp (insert + adapters) and AD fractions displayed. (PDF) [file pone.0217507.s002.pdf]

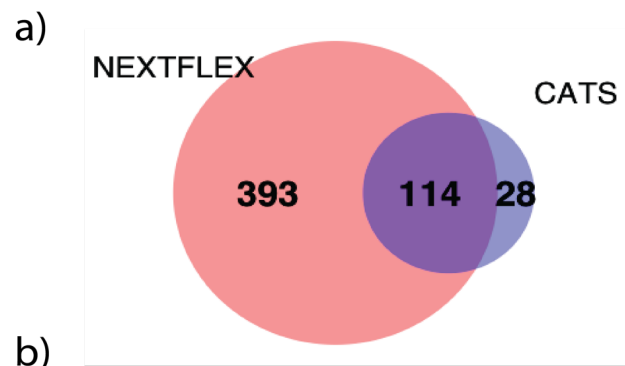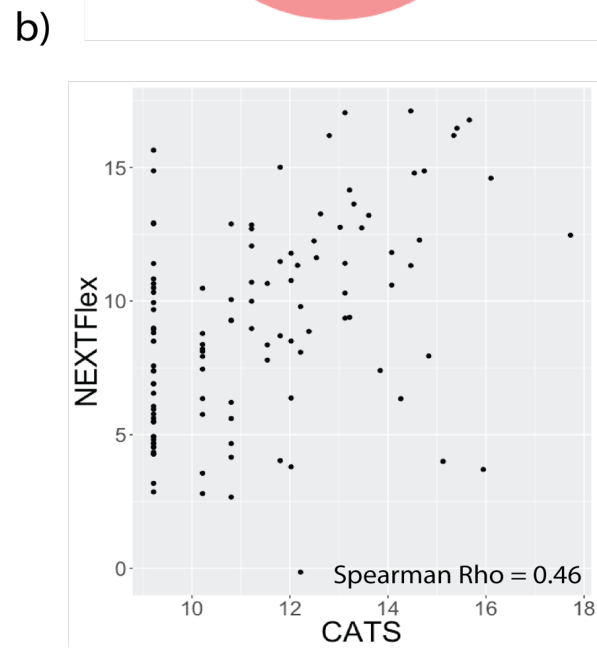

S3 Fig.

Supplement: S3 Fig — a) Venn diagram of the miRNAs identified in the ultracentrifugation samples showed 114 common and 421 exclusive miRNAs between the NEXTFlex and CATS preparations. b) Spearman correlation analysis of the ultracentrifugation miRNA expression derived from NEXTFlex and CATS preparations showed significant correlation, rho = 0.46. (PDF) [file pone.0217507.s003.pdf]

a)

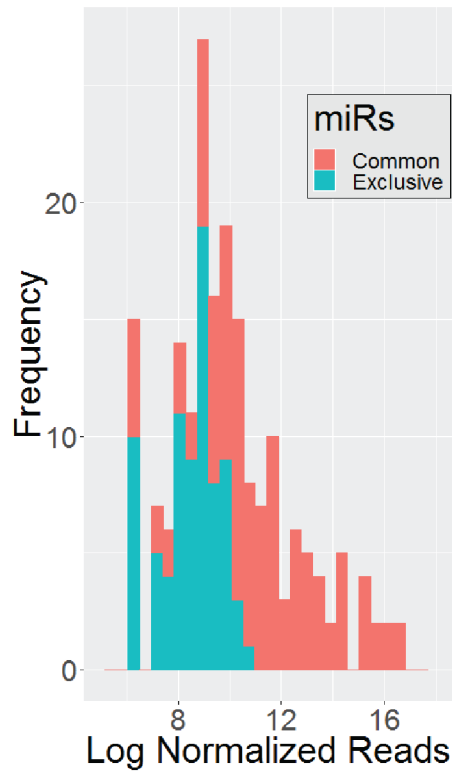

b)

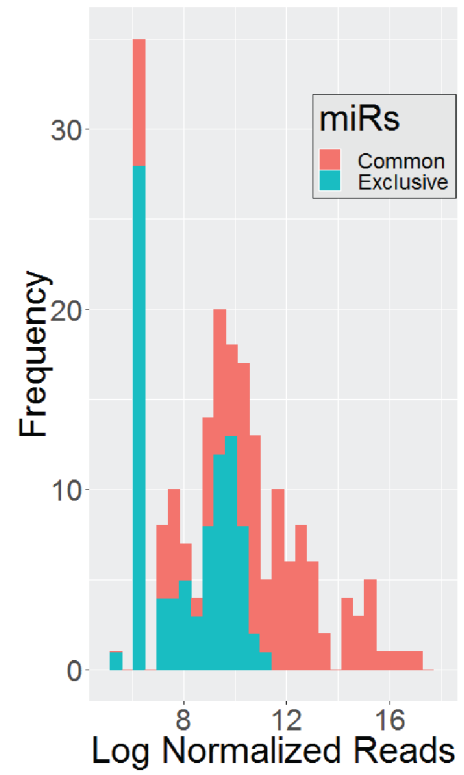

S4 Fig.

Supplement: S4 Fig — (PDF) [file pone.0217507.s004.pdf]

a)

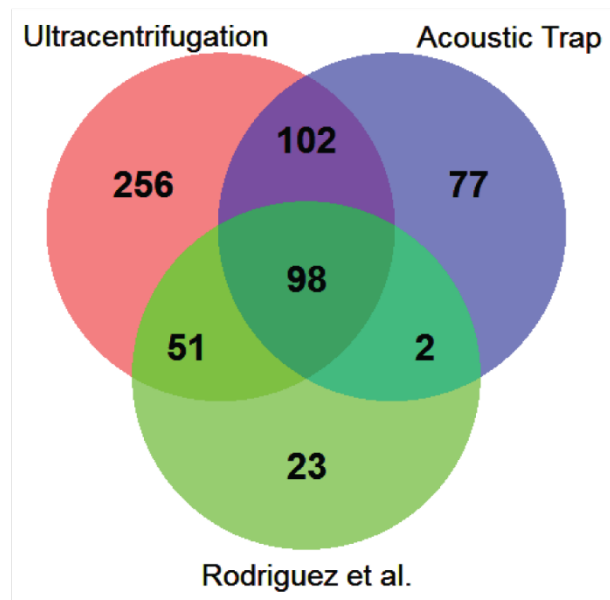

b)

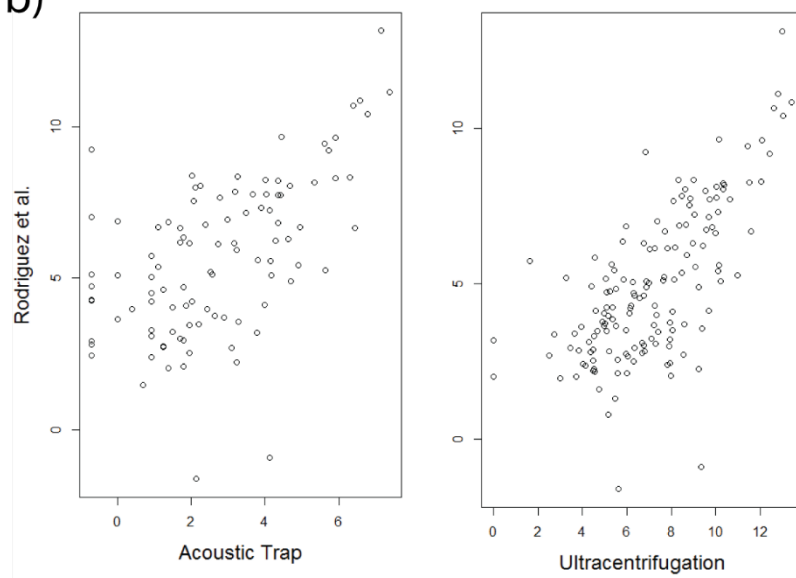

S5 Fig.

Supplement: S5 Fig — a) Analysis of common and exclusive miRNAs found in the acoustic trap, ultracentrifugation and Rodriguez et al. dataset b) Spearman correlation resulted in Rho of 0.64 and 0.72 respectively for acoustic trap, left and ultracentrifugation, right. (PDF) [file pone.0217507.s005.pdf]
