# Supplementary material for: A urinary extracellular vesicle microRNA biomarker discovery pipeline; from automated extracellular vesicle enrichment by acoustic trapping to microRNA sequencing
Source: PLoS One. 2019 May 29;14(5):e0217507. doi: 10.1371/journal.pone.0217507 (PMC6541292; doi:10.1371/journal.pone.0217507)
Supplement: S1 Table — (PDF) [file pone.0217507.s006.pdf]

Samples	Bioanalyzer RNA Concentration (pg/μL)
Ultracentrifugation	104±61 (n = 3)
Acoustic trap	90±8.7 (n = 2)
Blank	60±30 (n=3)

S1 Table
