# Supplementary material for: A urinary extracellular vesicle microRNA biomarker discovery pipeline; from automated extracellular vesicle enrichment by acoustic trapping to microRNA sequencing
Source: PLoS One. 2019 May 29;14(5):e0217507. doi: 10.1371/journal.pone.0217507 (PMC6541292; doi:10.1371/journal.pone.0217507)
Supplement: S2 Table — (PDF) [file pone.0217507.s007.pdf]

	**Ct Values**		
miRNAs	**UC**	**Mean Acoustic Trapped Replicates**	**Cel-miR-39 spike-in**
hsa-miR-21	23.0	30.3	NA
hsa-miR-24	25.3	33.2	NA
hsa-miR-16	26.1	33.4	NA
Cel-miR-39	29.5	27.8	27.6

S2 Table
