# Supplementary material for: A urinary extracellular vesicle microRNA biomarker discovery pipeline; from automated extracellular vesicle enrichment by acoustic trapping to microRNA sequencing
Source: PLoS One. 2019 May 29;14(5):e0217507. doi: 10.1371/journal.pone.0217507 (PMC6541292; doi:10.1371/journal.pone.0217507)
Supplement: S3 Table — (PDF) [file pone.0217507.s008.pdf]

Samples	Kit	PD Conc (pM)	Library Conc (pM)	Total Conc (pM)	% PD
Trap 1	NEXTflex	1657	4994	6651	25%
Trap 2	NEXTflex	1372	5327	6700	20%
UC	NEXTflex	1049	13272	14322	7%
Trap 1	CATS	1663	2089	3752	44%
Trap 2	CATS	596	8501	9098	7%
UC	CATS	572	43830	44402	1%

S3 Table
