# Supplementary material for: A urinary extracellular vesicle microRNA biomarker discovery pipeline; from automated extracellular vesicle enrichment by acoustic trapping to microRNA sequencing
Source: PLoS One. 2019 May 29;14(5):e0217507. doi: 10.1371/journal.pone.0217507 (PMC6541292; doi:10.1371/journal.pone.0217507)
Supplement: S4 Table — (PDF) [file pone.0217507.s009.pdf]

	NEXTFlex			CATS		
RNA Species	Acoustic Trap Replicate 1	Acoustic Trap Replicate 2	Ultracentrifguation	Acoustic Trap Replicate 1	Acoustic Trap Replicate 2	Ultracentrifguation
Low Complexity	4583	7933	17698	1409	4667	38979
miRNA	100746	82675	5564322	232	717	6567
rRNA	17688	26159	159607	5638	10476	73049
tRNA	27767	39822	3112482	406	1018	66604
piRNA	30306	24139	27300	448	965	727
snoRNA	126	248	26556	0	26	186
snRNA	221	171	3679	71	77	93
protein_coding	50627	40453	78164	440	963	3281
Others	19042	18015	501230	503	1263	18254
Total	1064023	1517825	11432141	596660	935621	743524

S4 Table
