# Supplementary material for: A urinary extracellular vesicle microRNA biomarker discovery pipeline; from automated extracellular vesicle enrichment by acoustic trapping to microRNA sequencing
Source: PLoS One. 2019 May 29;14(5):e0217507. doi: 10.1371/journal.pone.0217507 (PMC6541292; doi:10.1371/journal.pone.0217507)
Supplement: S5 Table — Yellow hightlighted cells represents miRNA found in AcT samples but not UC that were validated and blue highlights are differentially enriched miRNA in AcT samples used for validation. (PDF) [file pone.0217507.s010.pdf]

AcT1 ∩ UC	AcT2 ∩ UC	AcT1 ∩ AcT2	AcT1 - UC	AcT2 - UC	UC - (AcT1 ∪ AcT2)	
let-7a-2-5p	let-7a-2-5p	let-7a-2-5p	miR-10392-3p	miR-10392-3p	miR-369-3p	miR-4433a-5p
let-7a-5p	let-7a-5p	let-7a-5p	miR-103a-2-3p	miR-1178-3p	miR-218-1-3p	miR-940-3p
let-7b-5p	let-7b-5p	let-7b-5p	miR-10400-5p	miR-1181-3p	miR-320d-1-3p	miR-7-3-5p
let-7c-5p	let-7c-5p	let-7c-5p	miR-1260b-3p	miR-1183-3p	miR-320b	miR-944-3p
let-7d-3p	let-7d-3p	let-7d-3p	miR-1275-5p	miR-1202-3p	miR-548ad-5p	miR-1226-5p
let-7d-5p	let-7d-5p	let-7d-5p	miR-1281-3p	miR-1246	miR-548ab	miR-4767-3p
let-7e-5p	let-7e-5p	let-7e-5p	miR-1304-3p	miR-1306-5p	miR-548k	miR-1268a
let-7f-1-5p	let-7f-1-5p	let-7f-1-5p	miR-1307-5p	miR-1307-5p	miR-10a-3p	miR-1268b-5p
let-7f-5p	let-7f-2-5p	let-7f-5p	miR-182-3p	miR-142-5p	miR-618-5p	miR-1304-5p
let-7g-5p	let-7f-5p	let-7g-5p	miR-193a-3p	miR-151b-5p	miR-10527-5p	miR-200c-5p
let-7i-5p	let-7g-5p	let-7i-5p	miR-1976-3p	miR-1910-5p	miR-330-3p	miR-19b-2-3p
miR-100-5p	let-7i-3p	miR-100-5p	miR-3156-1-5p	miR-193a-3p	miR-652-3p	miR-9718-3p
miR-101-2-3p	let-7i-5p	miR-101-2-3p	miR-3187-3p	miR-1976-5p	miR-224-5p	miR-374b-3p
miR-101-3p	miR-100-5p	miR-10392-3p	miR-3652-5p	miR-19a-3p	miR-148a-5p	miR-331-5p
miR-103a-1-3p	miR-101-2-3p	miR-103a-3p	miR-4259-3p	miR-3180-5-3p	miR-7-1-3p	let-7a-3p
miR-103a-3p	miR-10399-5p	miR-10a-5p	miR-4302-3p	miR-33b-3p	miR-362-3p	miR-1284-5p
miR-106b-5p	miR-103a-3p	miR-10b-5p	miR-4314-3p	miR-3621-5p	miR-181a-2-3p	let-7e-3p
miR-10a-5p	miR-106a-5p	miR-12136-3p	miR-4468-3p	miR-378a-5p	miR-452-5p	miR-1468-5p
miR-10b-5p	miR-106b-3p	miR-12136-5p	miR-449b-5p	miR-3945-5p	miR-376c-3p	miR-4750-5p
miR-12136-3p	miR-107	miR-125a-5p	miR-4514-5p	miR-4283-2-5p	miR-181b-1-5p	miR-874-3p
miR-12136-5p	miR-107-3p	miR-125b-2-5p	miR-4516-5p	miR-4313-3p	miR-181b-2-5p	miR-1269a-3p
miR-1246-5p	miR-10a-5p	miR-125b-5p	miR-4653-3p	miR-4314-3p	miR-181c-5p	miR-1270
miR-125a-5p	miR-10b-3p	miR-1260a-5p	miR-4695-5p	miR-4316-5p	miR-4286-5p	miR-29b-2-5p
miR-125b-2-5p	miR-10b-5p	miR-128-1-3p	miR-4706-5p	miR-4448-3p	miR-487b-3p	miR-30c-2-5p
miR-125b-5p	miR-1180-3p	miR-128-2-3p	miR-4734-5p	miR-4514-5p	miR-28-5p	miR-6873-3p
miR-1260a-5p	miR-12136-3p	miR-1290-3p	miR-4778-5p	miR-4516-5p	miR-4636-5p	miR-184-3p
miR-1261-5p	miR-12136-5p	miR-1307-5p	miR-548a-3-3p	miR-4656-5p	miR-4636	miR-129-1-5p
miR-128-1-3p	miR-1225-5p	miR-135a-5p	miR-548at-5p	miR-486-5p	miR-4724-5p	miR-129-5p
miR-128-2-3p	miR-125a-3p	miR-146b-5p	miR-557-3p	miR-495-3p	miR-708-5p	miR-12136
miR-1290-3p	miR-125a-5p	miR-148a-3p	miR-6130-3p	miR-502-5p	miR-151b-3p	miR-7-1-5p
miR-135a-5p	miR-125b-2-5p	miR-151a-3p	miR-636-5p	miR-5094-3p	miR-4781-3p	miR-4699-5p
miR-135b-5p	miR-125b-5p	miR-151a-5p	miR-6506-5p	miR-548at-5p	miR-125b-2-3p	miR-4647
miR-140-3p	miR-1260a-5p	miR-152-3p	miR-6509-5p	miR-5739-3p	miR-4521-5p	miR-4284-5p
miR-141-3p	miR-1260b-5p	miR-15b-5p	miR-6773-5p	miR-618	miR-17-5p	miR-615-3p
miR-146b-5p	miR-128-1-3p	miR-191-5p	miR-6779-3p	miR-636-5p	miR-501-5p	miR-3620-5p
miR-148a-3p	miR-128-2-3p	miR-192-5p	miR-6782-5p	miR-658-5p	miR-656-3p	miR-203b-3p
miR-149-3p	miR-128-3p	miR-193a-3p	miR-6805-3p	miR-663b-3p	miR-34c-3p	miR-146b-3p
miR-151a-3p	miR-1290-3p	miR-193b-5p	miR-6819-5p	miR-6737-5p	miR-92b-3p	miR-370-3p
miR-151a-5p	miR-135a-2-3p	miR-194-1-5p	miR-6865-5p	miR-6756-3p	miR-4709-5p	miR-1285-1-5p
miR-152-3p	miR-135a-5p	miR-194-2-5p	miR-6870-5p	miR-6773-5p	miR-199b-3p	miR-301a-5p
miR-15b-5p	miR-146b-5p	miR-194-5p	miR-6894-5p	miR-6786-5p	miR-148b-3p	miR-3663-3p
miR-181b-5p	miR-148a-3p	miR-200a-3p	miR-7114-3p	miR-6805-3p	miR-625-3p	let-7a-3-5p
miR-181d-5p	miR-151a-3p	miR-200b-3p	miR-7161-3p	miR-7161-3p	miR-421-3p	miR-6821-5p
miR-186-5p	miR-151a-5p	miR-200c-3p	miR-8057-5p	miR-8087-3p	miR-16-2-3p	miR-138-1-3p
miR-191-5p	miR-152-3p	miR-203a-3p	miR-8063-3p		miR-181a-3p	miR-345-5p
miR-192-5p	miR-15b-5p	miR-204-3p	miR-9902-1-5p		miR-454-5p	miR-29b-1-5p
miR-193b-5p	miR-16-1-5p	miR-204-5p			miR-4324-3p	miR-3182-5p
miR-194-1-5p	miR-16-5p	miR-21-5p			miR-6827-3p	miR-136-5p
miR-194-2-5p	miR-181a-2-5p	miR-22-3p			miR-125b-1-3p	miR-505-5p
miR-194-5p	miR-181a-5p	miR-221-3p			miR-1306-3p	miR-718-5p
miR-196a-1-5p	miR-182-5p	miR-222-3p			let-7b-3p	miR-6855-5p
miR-196a-2-5p	miR-183-5p	miR-23a-3p			miR-1229-3p	miR-23a-5p
miR-196b-5p	miR-185-5p	miR-23b-3p			miR-130b-5p	miR-92a-2-5p
miR-200a-3p	miR-191-5p	miR-23c-3p			miR-664-5p	miR-23b-5p
miR-200b-3p	miR-192-5p	miR-24-1-3p			miR-1307-3p	miR-1268a-5p
miR-200c-3p	miR-193b-5p	miR-24-3p			miR-10399-3p	miR-24-1-5p

miR-203a-3p	miR-194-1-5p	miR-26a-2-5p			miR-2110-3p	miR-6790-3p
miR-204-3p	miR-194-2-5p	miR-26a-5p			miR-378c-5p	miR-3613-5p
miR-204-5p	miR-194-5p	miR-26b-5p			miR-378f-3p	miR-506-3p
miR-205-5p	miR-196a-5p	miR-27a-3p			miR-1270-5p	miR-877-5p
miR-20a-5p	miR-199a-2-3p	miR-27b-3p			miR-653-3p	miR-132-3p
miR-21-5p	miR-200a-3p	miR-28-3p			miR-5588-5p	miR-1236-5p
miR-218-5p	miR-200b-3p	miR-29a-3p			miR-149-5p	miR-4758-5p
miR-22-3p	miR-200b-5p	miR-30a-3p			miR-146a-5p	miR-1343-5p
miR-221-3p	miR-200c-3p	miR-30a-5p			miR-6745-3p	miR-3074-5p
miR-222-3p	miR-203a-3p	miR-30b-5p			miR-504-5p	miR-582-3p
miR-23a-3p	miR-204-3p	miR-30c-1-5p			miR-6131-3p	miR-124-1-3p
miR-23b-3p	miR-204-5p	miR-30c-5p			miR-27b-5p	miR-193a-5p
miR-23c-3p	miR-206	miR-30d-5p			miR-485-5p	miR-18a-3p
miR-24-1-3p	miR-21-5p	miR-30e-3p			miR-605-3p	miR-500b-5p
miR-24-2-5p	miR-2110-5p	miR-30e-5p			miR-574-5p	miR-500a-5p
miR-24-3p	miR-215-5p	miR-3178-5p			miR-508-3p	miR-216a-5p
miR-26a-2-5p	miR-22-3p	miR-320a-3p			miR-891a-5p	miR-365b-3p
miR-26a-5p	miR-221-3p	miR-320b-1-3p			miR-29b-1-3p	miR-590-3p
miR-26b-5p	miR-221-5p	miR-320b-2-3p			miR-424-5p	miR-514a-1-5p
miR-27a-3p	miR-222-3p	miR-320e-3p			miR-210-5p	miR-4802-3p
miR-27a-5p	miR-23a-3p	miR-335-3p			miR-3173-5p	miR-598-3p
miR-27b-3p	miR-23b-3p	miR-335-5p			miR-92b-5p	miR-888-5p
miR-28-3p	miR-23c-3p	miR-34b-3p			miR-34a-5p	miR-508-5p
miR-29a-3p	miR-24-1-3p	miR-363-3p			miR-6876-5p	miR-509-2-5p
miR-29c-3p	miR-24-3p	miR-3648-2-5p			miR-1255a-5p	miR-195-5p
miR-30a-3p	miR-25-3p	miR-374b-5p			miR-30c-1-3p	miR-15a-5p
miR-30a-5p	miR-25-5p	miR-375-3p			miR-647-3p	miR-16-2-5p
miR-30b-5p	miR-26a-2-3p	miR-379-5p			miR-342-5p	miR-34c-5p
miR-30c-1-5p	miR-26a-2-5p	miR-423-3p			miR-629-5p	miR-493-5p
miR-30c-2-3p	miR-26a-5p	miR-423-5p			miR-4538-3p	miR-5004-5p
miR-30c-5p	miR-26b-5p	miR-429			miR-92a-1-5p	miR-1843-5p
miR-30d-5p	miR-27a-3p	miR-4314-3p			miR-22-5p	miR-135a-2-5p
miR-30e-3p	miR-27b-3p	miR-4443-5p			miR-542-3p	miR-664a-3p
miR-30e-5p	miR-28-3p	miR-4488-5p			miR-500b-3p	miR-92a-2-3p
miR-3178-5p	miR-29a-3p	miR-4508-5p			miR-934-3p	miR-3065-5p
miR-320a-3p	miR-30a-3p	miR-4514-5p			miR-26a-1-5p	miR-12125-3p
miR-320b-1-3p	miR-30a-5p	miR-4516-5p			miR-340-5p	miR-3664-3p
miR-320b-2-3p	miR-30b-5p	miR-501-3p			miR-9-3p	miR-340-3p
miR-320e-3p	miR-30c-1-5p	miR-532-5p			miR-499a-5p	miR-338-3p
miR-335-3p	miR-30c-5p	miR-548at-5p			miR-155-5p	miR-125b-1-5p
miR-335-5p	miR-30d-5p	miR-628-3p			miR-4662a-5p	miR-671-3p
miR-342-3p	miR-30e-3p	miR-636-5p			miR-421	miR-1237-3p
miR-34b-3p	miR-30e-5p	miR-6773-5p			miR-95-3p	miR-151b
miR-362-5p	miR-31-5p	miR-6805-3p			miR-513a-2-5p	miR-6771-5p
miR-363-3p	miR-3178-5p	miR-6816-5p			miR-197-3p	miR-187-3p
miR-3648-2-5p	miR-320a-3p	miR-7161-3p			miR-200a-5p	miR-6787-3p
miR-365a-3p	miR-320b-1-3p	miR-760-5p			miR-556-5p	miR-1843-3p
miR-374a-5p	miR-320b-2-3p	miR-7703-5p			let-7a-1-5p	miR-4742-3p
miR-374b-5p	miR-320c-1-3p	miR-9-1-5p			miR-1301-3p	miR-942-5p
miR-375-3p	miR-320e-3p	miR-92a-1-3p			miR-455-3p	miR-589-5p
miR-379-5p	miR-335-3p	miR-92a-3p			miR-6872-3p	miR-769-5p
miR-423-3p	miR-335-5p	miR-98-5p			miR-628-5p	miR-4510-5p
miR-423-5p	miR-338-5p	miR-99a-5p			miR-30b-3p	miR-6875-5p
miR-424-3p	miR-339-3p	miR-99b-3p			miR-6843-3p	miR-6734-5p
miR-425-3p	miR-34b-3p	miR-99b-5p			miR-374a-3p	miR-190a-5p
miR-429	miR-361-3p				miR-576-5p	miR-509-3p
miR-429-3p	miR-361-5p				miR-514a-3p	miR-891b-5p
miR-4429-5p	miR-3615-3p				miR-450a-1-3p	miR-744-5p
miR-4443-5p	miR-363-3p				miR-450a-2-3p	miR-7-5p
miR-4455-5p	miR-3648-2-5p				miR-509-2-3p	miR-1-3p

miR-4485-3p	miR-365a-5p				miR-450b-5p	miR-206-3p
miR-4488-5p	miR-374b-5p				miR-450a-1-5p	miR-184
miR-4508-5p	miR-375-3p				miR-548am-5p	miR-6515-5p
miR-484	miR-378a-3p				miR-20b-5p	miR-1291-3p
miR-484-5p	miR-378i-5p				miR-21-3p	miR-6721-5p
miR-489-3p	miR-379-5p				miR-708-3p	miR-6508-3p
miR-500a-3p	miR-382-5p				miR-194-2-3p	miR-664b-5p
miR-501-3p	miR-3960-3p				let-7a-3-3p	miR-4450
miR-502-3p	miR-423-3p				miR-32-3p	miR-4536-2-5p
miR-503-5p	miR-423-5p				miR-6750-3p	miR-412-5p
miR-5100-3p	miR-425-5p				miR-4485-5p	miR-223-3p
miR-532-5p	miR-429				miR-1233-2-5p	miR-934
miR-6126-5p	miR-4443-5p				miR-3144-5p	miR-934-5p
miR-628-3p	miR-4454-5p				miR-892a-3p	miR-4796-5p
miR-641-5p	miR-4488-5p				miR-3934-5p	miR-4504
miR-653-5p	miR-4508-5p				miR-3065-3p	miR-19b-3p
miR-6816-5p	miR-451a				miR-328-3p	miR-582-5p
miR-760-5p	miR-454-3p				miR-218-2-5p	miR-6866-5p
miR-7703-5p	miR-501-3p				miR-130a-3p	miR-4662b-5p
miR-9-1-5p	miR-505-3p				miR-676-3p	miR-1295a-3p
miR-9-5p	miR-532-3p				miR-130b-3p	miR-1296-5p
miR-92a-1-3p	miR-532-5p				miR-140-5p	miR-584-5p
miR-92a-3p	miR-574-3p				miR-4800-3p	miR-513b-5p
miR-93-5p	miR-628-3p				miR-30d-3p	miR-6883-3p
miR-98-5p	miR-660-5p				miR-195-3p	miR-3124-5p
miR-99a-5p	miR-664-3p				miR-194-3p	miR-514a-1-3p
miR-99b-3p	miR-6803-3p				miR-6747-3p	miR-4443
miR-99b-5p	miR-6816-5p				miR-181a-1-3p	miR-6740-5p
	miR-760-5p				miR-323b-3p	miR-2110
	miR-7703-5p				miR-199b-5p	miR-1303-3p
	miR-7975-3p				miR-8078-5p	miR-1303
	miR-7977-5p				miR-339-5p	miR-651-5p
	miR-9-1-3p				miR-126-3p	miR-122b-5p
	miR-9-1-5p				miR-6511b-1-3p	miR-96-5p
	miR-92a-1-3p				miR-3605-3p	miR-450a-5p
	miR-92a-3p				miR-26b-3p	
	miR-98-5p				miR-15b-3p	
	miR-99a-5p				miR-409-3p	
	miR-99b-3p				miR-3605-5p	
	miR-99b-5p				miR-1234-5p	

S5 Table
