# Supplementary material for: A urinary extracellular vesicle microRNA biomarker discovery pipeline; from automated extracellular vesicle enrichment by acoustic trapping to microRNA sequencing
Source: PLoS One. 2019 May 29;14(5):e0217507. doi: 10.1371/journal.pone.0217507 (PMC6541292; doi:10.1371/journal.pone.0217507)
Supplement: S6 Table — (PDF) [file pone.0217507.s011.pdf]

miR	UC	AcT replicate 1	AcT replicate 2	miR	UC	AcT replicate 1	AcT replicate 2	miR	UC	AcT replicate 1	AcT replicate 2
miR-30a-5p	674176	1021	412	miR-26b-5p	6966	42	71	miR-15b-5p	1085	3	1
let-7a-5p	466628	900	855	miR-103a-3p	6768	1	15	miR-141-3p	1084	5	0
miR-10b-5p	445134	1273	1278	miR-423-3p	6008	36	15	miR-10a-3p	1015	0	0
let-7b-5p	368595	1397	1817	miR-22-3p	5760	20	20	miR-502-3p	1012	8	0
miR-10a-5p	297936	633	569	miR-28-3p	5516	16	3	miR-135a-2-3p	1010	0	6
let-7a-2-5p	246977	525	529	miR-320b-1-3p	5469	10	30	miR-151b-3p	986	0	0
let-7f-5p	246240	327	292	miR-23c-3p	5333	2	23	miR-196a-2-5p	981	13	0
miR-26a-5p	175650	332	415	miR-30b-5p	5316	22	14	miR-103a-1-3p	981	16	0
miR-30d-5p	168972	551	531	miR-1260a-5p	5227	25	88	miR-196a-1-5p	975	8	0
let-7g-5p	108606	166	115	miR-29a-3p	5118	41	3	miR-125b-2-3p	969	0	0
miR-30c-1-5p	98888	334	494	miR-186-5p	4762	6	0	miR-148b-3p	953	0	0
miR-21-5p	98600	464	281	miR-146b-5p	4707	15	34	miR-181a-5p	939	0	1
let-7c-5p	93231	218	332	miR-95-3p	4491	0	0	miR-505-3p	915	0	20
let-7f-1-5p	81646	132	120	miR-9-1-5p	4327	7	4	miR-135a-5p	911	14	23
miR-375-3p	80072	198	133	miR-183-5p	4234	0	2	miR-361-3p	896	0	1
miR-125a-5p	59986	262	303	miR-151a-3p	4159	28	24	let-7a-1-5p	863	0	0
miR-200c-3p	41801	108	47	miR-335-5p	4089	1	6	miR-205-5p	863	7	0
let-7e-5p	32442	81	75	miR-760-5p	3914	262	93	miR-361-5p	859	0	11
miR-99b-5p	31185	71	39	miR-30c-2-3p	3516	11	0	miR-92b-3p	859	0	0
miR-92a-3p	31079	117	100	miR-93-5p	3363	26	0	miR-29c-3p	812	5	0
miR-200b-3p	27125	73	57	miR-30e-3p	3248	9	23	miR-500a-3p	803	7	0
miR-99a-5p	25634	35	56	miR-429	3108	16	2	miR-12136-5p	766	26	15
miR-30a-3p	25047	86	84	miR-363-3p	3097	12	1	miR-16-1-5p	731	0	6
miR-27b-3p	24959	136	133	miR-92a-1-3p	2981	28	3	miR-31-5p	704	0	5
miR-423-5p	24338	45	53	miR-200b-5p	2928	0	8	miR-149-5p	693	0	0
miR-191-5p	22915	14	65	miR-24-3p	2845	18	10	miR-128-1-3p	628	11	6
miR-204-5p	22536	167	253	miR-128-3p	2818	0	1	miR-450b-5p	597	0	0
miR-30e-5p	22014	613	644	miR-194-5p	2798	48	40	miR-135b-5p	577	3	0
miR-98-5p	18637	97	60	miR-28-5p	2708	0	0	miR-130b-5p	576	0	0
miR-148a-3p	16885	88	79	miR-501-3p	2507	2	3	miR-10b-3p	572	0	12
let-7d-5p	16421	34	31	miR-222-3p	2505	22	26	miR-200a-5p	558	0	0
miR-151a-5p	16330	71	38	miR-25-3p	2215	0	6	miR-29b-1-3p	557	0	0
miR-26a-2-5p	16038	87	47	miR-532-5p	2165	16	9	miR-7-5p	553	0	0
miR-200a-3p	14414	12	10	miR-140-3p	2056	2	0	miR-660-5p	553	0	12
let-7i-5p	14086	9	7	miR-27a-3p	2028	4	2	miR-374b-5p	547	13	19
miR-30c-5p	12434	49	96	miR-16-5p	1670	0	14	miR-193a-5p	544	0	0
miR-23b-3p	11901	14	39	miR-24-1-3p	1653	15	2	miR-484-5p	528	4	0
miR-23a-3p	11659	74	51	miR-378a-3p	1613	0	1	miR-181a-2-3p	521	0	0
miR-320a-3p	11385	51	16	miR-574-3p	1535	0	3	miR-146a-5p	496	0	0
miR-125b-5p	10383	32	19	miR-340-5p	1482	0	0	miR-374a-5p	490	5	0
miR-100-5p	10378	125	96	miR-181a-2-5p	1454	0	24	miR-9-1-3p	489	0	4
miR-101-2-3p	9416	46	18	miR-196a-5p	1420	0	14	miR-7-3-5p	474	0	0
miR-125b-2-5p	8979	27	76	miR-425-5p	1409	0	1	miR-1180-3p	468	0	5
let-7d-3p	8928	47	79	miR-424-3p	1373	2	0	miR-542-3p	456	0	0
let-7f-2-5p	8508	0	28	miR-342-3p	1198	9	0	miR-196b-5p	453	9	0
miR-192-5p	8469	46	77	miR-224-5p	1193	0	0	miR-340-3p	438	0	0
miR-182-5p	8287	0	15	miR-429-3p	1192	4	0	miR-320c-1-3p	429	0	9
miR-203a-3p	7789	168	40	miR-221-3p	1140	8	23	miR-7977-5p	429	0	18
miR-194-2-5p	7384	57	48	miR-194-1-5p	1096	26	24	miR-197-3p	421	0	0

miR	UC	AcT replicate 1	AcT replicate 2	miR	UC	AcT replicate 1	AcT replicate 2	miR	UC	AcT replicate 1	AcT replicate 2
miR-204-3p	402	8	4	miR-132-3p	179	0	0	miR-589-5p	88	0	0
miR-181b-5p	395	8	0	miR-99b-3p	177	12	11	miR-1226-5p	88	0	0
miR-484	393	7	0	miR-34b-3p	175	13	7	miR-9-3p	87	0	0
miR-215-5p	392	0	7	miR-194-3p	172	0	0	miR-92b-5p	87	0	0
miR-374a-3p	392	0	0	miR-7975-3p	169	0	13	miR-513b-5p	87	0	0
miR-450a-1-5p	363	0	0	miR-628-3p	161	8	7	miR-1303	87	0	0
miR-652-3p	347	0	0	miR-181d-5p	160	1	0	miR-30c-2-5p	84	0	0
miR-9-5p	347	1	0	miR-584-5p	160	0	0	miR-106a-5p	83	0	11
miR-101-3p	338	12	0	miR-106b-3p	159	0	1	miR-454-3p	82	0	10
miR-499a-5p	333	0	0	miR-125a-3p	154	0	1	miR-328-3p	82	0	0
miR-22-5p	318	0	0	miR-128-2-3p	154	5	9	miR-17-5p	82	0	0
miR-362-5p	317	10	0	miR-574-5p	154	0	0	miR-625-3p	80	0	0
miR-1260b-5p	316	0	8	miR-1229-3p	152	0	0	miR-365b-3p	79	0	0
miR-185-5p	307	0	2	miR-421	149	0	0	miR-3178-5p	79	18	8
miR-2110-5p	304	0	1	miR-1246-5p	148	1	0	miR-1270	79	0	0
miR-455-3p	290	0	0	miR-1261-5p	148	13	0	miR-199b-5p	78	0	0
miR-664-5p	288	0	0	miR-1301-3p	147	0	0	miR-1290-3p	77	11	20
miR-4286-5p	281	0	0	miR-184	145	0	0	miR-582-3p	75	0	0
miR-335-3p	280	10	7	miR-421-3p	145	0	0	miR-15a-5p	75	0	0
miR-4662a-5p	278	0	0	miR-92a-1-5p	144	0	0	miR-3065-3p	74	0	0
miR-450a-5p	278	0	0	miR-503-5p	142	4	0	miR-409-3p	74	0	0
miR-26a-1-5p	277	0	0	miR-514a-1-5p	136	0	0	miR-193b-5p	73	8	1
miR-379-5p	277	16	24	miR-30b-3p	133	0	0	miR-4443-5p	72	9	1
miR-12136-3p	272	19	47	miR-615-3p	132	0	0	miR-1468-5p	72	0	0
miR-339-5p	272	0	0	miR-452-5p	128	0	0	miR-4750-5p	72	0	0
miR-34a-5p	268	0	0	miR-4488-5p	126	4	1	miR-330-3p	71	0	0
miR-424-5p	267	0	0	miR-888-5p	120	0	0	miR-1268a	71	0	0
miR-338-5p	263	0	2	let-7b-3p	113	0	0	miR-556-5p	70	0	0
miR-218-5p	263	14	0	miR-664-3p	113	0	7	miR-3615-3p	70	0	1
miR-769-5p	250	0	0	miR-3605-3p	112	0	0	miR-20a-5p	70	4	0
miR-598-3p	243	0	0	miR-5588-5p	110	0	0	miR-509-3p	69	0	0
miR-1843-3p	239	0	0	miR-514a-3p	109	0	0	miR-454-5p	69	0	0
miR-487b-3p	235	0	0	miR-21-3p	108	0	0	miR-653-3p	69	0	0
miR-629-5p	234	0	0	miR-10527-5p	107	0	0	miR-27b-5p	69	0	0
miR-891a-5p	224	0	0	miR-342-5p	107	0	0	miR-3613-5p	69	0	0
miR-181b-1-5p	221	0	0	miR-369-3p	106	0	0	miR-582-5p	69	0	0
let-7a-3-5p	219	0	0	miR-194-2-3p	99	0	0	miR-489-3p	68	10	0
miR-152-3p	209	5	10	miR-12136	99	0	0	miR-618-5p	66	0	0
miR-744-5p	209	0	0	miR-653-5p	99	1	0	miR-3065-5p	65	0	0
miR-532-3p	203	0	10	miR-151b	98	0	0	miR-4636	63	0	0
miR-1307-3p	201	0	0	miR-96-5p	98	0	0	miR-140-5p	62	0	0
miR-508-3p	201	0	0	miR-218-1-3p	97	0	0	miR-107-3p	62	0	3
miR-4455-5p	200	2	0	miR-4742-3p	95	0	0	let-7a-3p	62	0	0
miR-16-2-3p	200	0	0	miR-148a-5p	94	0	0	miR-7-1-5p	62	0	0
miR-339-3p	194	0	1	miR-874-3p	94	0	0	miR-892a-3p	61	0	0
miR-218-2-5p	190	0	0	miR-23b-5p	93	0	0	miR-509-2-3p	60	0	0
miR-106b-5p	189	4	0	miR-30c-1-3p	91	0	0	miR-4521-5p	60	0	0
miR-155-5p	186	0	0	miR-425-3p	91	5	0	miR-708-3p	58	0	0
miR-126-3p	183	0	0	miR-221-5p	91	0	1	miR-493-5p	57	0	0

miR	UC	AcT replicate 1	AcT replicate 2	miR	UC	AcT replicate 1	AcT replicate 2	miR	UC	AcT replicate 1	AcT replicate 2
miR-20b-5p	57	0	0	miR-1343-5p	15	0	0	miR-450a-2-3p	38	0	0
miR-374b-3p	57	0	0	miR-19b-3p	15	0	0	miR-4536-2-5p	38	0	0
miR-877-5p	56	0	0	miR-500b-3p	14	0	0	miR-26a-2-3p	38	0	18
miR-664a-3p	55	0	0	miR-135a-2-5p	13	0	0	miR-4443	38	0	0
miR-2110-3p	53	0	0	miR-2110	13	0	0	miR-6515-5p	37	0	0
miR-203b-3p	53	0	0	miR-6721-5p	12	0	0	miR-19b-2-3p	37	0	0
miR-190a-5p	52	0	0	miR-181a-3p	12	0	0	miR-184-3p	37	0	0
miR-1234-5p	52	0	0	miR-3620-5p	11	0	0	miR-320b-2-3p	36	17	3
miR-130a-3p	51	0	0	miR-4324-3p	10	0	0	miR-34c-3p	36	0	0
miR-6734-5p	51	0	0	miR-3664-3p	10	0	0	miR-323b-3p	36	0	0
miR-3182-5p	51	0	0	miR-4758-5p	10	0	0	miR-138-1-3p	36	0	0
miR-223-3p	51	0	0	miR-1306-3p	9	0	0	miR-4796-5p	36	0	0
miR-504-5p	50	0	0	miR-378f-3p	9	0	0	miR-1237-3p	35	0	0
miR-15b-3p	50	0	0	miR-509-2-5p	8	0	0	miR-1285-1-5p	35	0	0
miR-548ab	49	0	0	miR-92a-2-5p	8	0	0	miR-1-3p	34	0	0
miR-382-5p	49	0	11	miR-656-3p	7	0	0	miR-6827-3p	34	0	0
miR-195-5p	48	0	0	miR-590-3p	5	0	0	miR-4647	34	0	0
miR-641-5p	47	3	0	miR-107	5	0	5	miR-1295a-3p	34	0	0
miR-4538-3p	47	0	0	miR-18a-3p	4	0	0	miR-216a-5p	33	0	0
miR-16-2-5p	46	0	0	miR-1236-5p	4	0	0	miR-664b-5p	33	0	0
miR-671-3p	46	0	0	miR-4709-5p	3	0	0	miR-412-5p	33	0	0
miR-331-5p	45	0	0	miR-4433a-5p	3	0	0	miR-505-5p	33	0	0
miR-4284-5p	45	0	0	miR-3124-5p	3	0	0	miR-365a-5p	32	0	1
miR-32-3p	44	0	0	miR-320e-3p	2	3	2	miR-199b-3p	32	0	0
miR-4508-5p	44	10	17	miR-365a-3p	2	5	0	miR-370-3p	32	0	0
miR-338-3p	44	0	0	miR-513a-2-5p	2	0	0	miR-6866-5p	32	0	0
miR-26b-3p	44	0	0	miR-3934-5p	2	0	0	miR-362-3p	31	0	0
miR-29b-1-5p	44	0	0	miR-1291-3p	2	0	0	miR-4800-3p	31	0	0
miR-6883-3p	44	0	0	miR-6131-3p	2	0	0	miR-942-5p	31	0	0
miR-12125-3p	43	0	0	miR-4454-5p	2	0	2	miR-378c-5p	31	0	0
miR-6787-3p	43	0	0	miR-3663-3p	2	0	0	miR-1843-5p	31	0	0
miR-136-5p	43	0	0	miR-6790-3p	2	0	0	miR-6511b-1-3p	31	0	0
miR-718-5p	43	0	0	miR-4662b-5p	2	0	0	miR-6821-5p	31	0	0
miR-934	43	0	0	miR-1296-5p	2	0	0	miR-3074-5p	31	0	0
miR-195-3p	42	0	0	miR-1303-3p	2	0	0	miR-6816-5p	30	687	536
miR-501-5p	42	0	0	miR-4429-5p	1	3	0	miR-301a-5p	30	0	0
miR-4802-3p	41	0	0	miR-376c-3p	1	0	0	miR-548ad-5p	29	0	0
miR-628-5p	41	0	0	miR-181b-2-5p	1	0	0	miR-508-5p	29	0	0
miR-30d-3p	41	0	0	miR-4636-5p	1	0	0	miR-6843-3p	29	0	0
miR-891b-5p	41	0	0	miR-6126-5p	1	5	0	miR-676-3p	29	0	0
miR-6508-3p	41	0	0	miR-500b-5p	1	0	0	miR-199a-2-3p	29	0	1
miR-485-5p	41	0	0	miR-5004-5p	1	0	0	miR-1270-5p	29	0	0
miR-647-3p	40	0	0	miR-6876-5p	1	0	0	miR-187-3p	29	0	0
miR-27a-5p	40	4	0	miR-149-3p	1	4	0	miR-1304-5p	29	0	0
miR-6872-3p	40	0	0	miR-934-3p	1	0	0	miR-514a-1-3p	29	0	0
miR-451a	39	0	1	miR-5100-3p	1	5	0	miR-6745-3p	28	0	0
miR-4699-5p	39	0	0	let-7a-3-3p	1	0	0	miR-210-5p	28	0	0
miR-146b-3p	39	0	0	miR-6750-3p	1	0	0	miR-3173-5p	28	0	0
miR-320b	38	0	0	miR-1233-2-5p	1	0	0	miR-181a-1-3p	28	0	0

miR	UC	AcT replicate 1	AcT replicate 2	miR	UC	AcT replicate 1	AcT replicate 2	miR	UC	AcT replicate 1	AcT replicate 2
miR-130b-3p	1	0	0	miR-3605-5p	28	0	0	miR-7114-3p	0	5	0
miR-4510-5p	1	0	0	miR-1284-5p	28	0	0	miR-4734-5p	0	2	0
miR-10399-5p	1	0	1	miR-122b-5p	28	0	0	miR-1281-3p	0	1	0
miR-6771-5p	1	0	0	miR-320d-1-3p	27	0	0	miR-1976-3p	0	4	0
miR-6747-3p	1	0	0	miR-548k	27	0	0	miR-4695-5p	0	3	0
miR-8078-5p	1	0	0	miR-25-5p	27	0	1	miR-486-5p	0	0	11
miR-940-3p	1	0	0	miR-4485-5p	27	0	0	miR-5739-3p	0	0	4
miR-4767-3p	1	0	0	miR-6803-3p	27	0	10	miR-1183-3p	0	0	3
miR-3648-2-5p	1	4	21	let-7e-3p	27	0	0	miR-1181-3p	0	0	4
miR-9718-3p	1	0	0	miR-1269a-3p	27	0	0	miR-19a-3p	0	0	1
miR-129-1-5p	1	0	0	miR-181c-5p	26	0	0	miR-1307-5p	0	1	1
miR-345-5p	1	0	0	miR-500a-5p	26	0	0	miR-10392-3p	0	18	10
miR-7703-5p	1	1	16	miR-4781-3p	26	0	0	miR-4706-5p	0	20	0
miR-6855-5p	1	0	0	miR-6873-3p	26	0	0	miR-1260b-3p	0	9	0
miR-1268a-5p	1	0	0	miR-129-5p	26	0	0	miR-4468-3p	0	1	0
let-7i-3p	1	0	1	miR-24-2-5p	26	5	0	miR-6782-5p	0	6	0
miR-1225-5p	1	0	1	miR-934-5p	26	0	0	miR-4516-5p	0	18	11
miR-557-3p	0	6	0	miR-708-5p	25	0	0	miR-6773-5p	0	20	10
miR-618	0	0	4	miR-124-1-3p	25	0	0	miR-6865-5p	0	8	0
miR-495-3p	0	0	12	miR-3960-3p	25	0	16	miR-8057-5p	0	12	0
miR-8087-3p	0	0	12	miR-4504	25	0	0	miR-10400-5p	0	4	0
miR-1246	0	0	15	miR-1255a-5p	24	0	0	miR-4259-3p	0	12	0
miR-4313-3p	0	0	9	miR-1268b-5p	24	0	0	miR-3187-3p	0	12	0
miR-1976-5p	0	0	7	miR-4724-5p	23	0	0	miR-6819-5p	0	7	0
miR-5094-3p	0	0	12	miR-6875-5p	23	0	0	miR-3652-5p	0	7	0
miR-4283-2-5p	0	0	8	miR-605-3p	23	0	0	miR-6509-5p	0	10	0
miR-3180-5-3p	0	0	15	miR-4485-3p	22	15	0	miR-502-5p	0	0	16
miR-1306-5p	0	0	1	miR-34c-5p	22	0	0	miR-1202-3p	0	0	1
miR-1910-5p	0	0	1	miR-548am-5p	22	0	0	miR-4316-5p	0	0	1
miR-142-5p	0	0	10	miR-206	22	0	9	miR-6737-5p	0	0	8
miR-3621-5p	0	0	7	miR-944-3p	22	0	0	miR-4448-3p	0	0	17
miR-33b-3p	0	0	3	miR-10399-3p	21	0	0	miR-9902-1-5p	0	11	0
miR-6786-5p	0	0	1	miR-200c-5p	21	0	0	miR-1275-5p	0	14	0
miR-151b-5p	0	0	4	miR-29b-2-5p	21	0	0	miR-1304-3p	0	4	0
miR-658-5p	0	0	1	miR-23a-5p	21	0	0	miR-193a-3p	0	8	3
miR-1178-3p	0	0	13	miR-125b-1-3p	20	0	0	miR-4778-5p	0	1	0
miR-378a-5p	0	0	1	miR-125b-1-5p	20	0	0	miR-3156-1-5p	0	8	0
miR-4656-5p	0	0	9	miR-651-5p	20	0	0	miR-6894-5p	0	4	0
miR-4302-3p	0	1	0	miR-7-1-3p	19	0	0	miR-548at-5p	0	7	43
miR-103a-2-3p	0	6	0	miR-576-5p	19	0	0	miR-6506-5p	0	6	0
miR-4514-5p	0	34	14	miR-3144-5p	19	0	0	miR-449b-5p	0	6	0
miR-8063-3p	0	7	0	miR-206-3p	19	0	0	miR-4314-3p	0	23	14
miR-7161-3p	0	7	30	miR-4450	19	0	0	miR-636-5p	0	1	1
miR-4653-3p	0	2	0	miR-506-3p	19	0	0	miR-6779-3p	0	1	0
miR-6130-3p	0	1	0	miR-6740-5p	19	0	0	miR-6756-3p	0	0	1
miR-6870-5p	0	8	0	miR-450a-1-3p	17	0	0	miR-3945-5p	0	0	7
miR-182-3p	0	20	0	miR-378i-5p	17	0	1	miR-663b-3p	0	0	1
miR-6805-3p	0	48	5	miR-24-1-5p	17	0	0				
miR-548a-3-3p	0	6	0	miR-92a-2-3p	16	0	0				

S6 Table
