# Supplementary material for: A urinary extracellular vesicle microRNA biomarker discovery pipeline; from automated extracellular vesicle enrichment by acoustic trapping to microRNA sequencing
Source: PLoS One. 2019 May 29;14(5):e0217507. doi: 10.1371/journal.pone.0217507 (PMC6541292; doi:10.1371/journal.pone.0217507)
Supplement: S7 Table — (PDF) [file pone.0217507.s012.pdf]

miRNA	C1	C2	C3	C10	C5	C6	C7	C8	C9
hsa-let-7a-5p	43445.1	61968.5	30341.9	33693.9	21264.6	33524.5	22415.5	33367.6	18207.3
hsa-let-7a-5p	43525.4	62056.7	30400.8	33767.3	21303.2	33595.4	22512	33404.9	18223.9
hsa-let-7a-5p	44214.9	63094.2	31042.2	34961.8	22027.7	34515.1	23073.8	34704.5	18742.8
hsa-let-7b-5p	104060.1	147858.8	73869.9	47914.8	45375.9	78744	61529.4	67738.4	49346.1
hsa-let-7c-5p	23893.7	32887.8	12243.5	9790	9597.1	17335.7	12424.8	19888.1	8955.1
hsa-let-7d-3p	247.3	317.3	238.4	286	192.9	415.5	303.2	259.6	197.4
hsa-let-7d-5p	1815.5	2363.5	1274.5	1186.2	865.4	1683.3	819.3	1279	888.3
hsa-let-7e-5p	3963.7	5281	3630.9	1877.2	2101.9	4563.9	3505.5	3869.1	2711.3
hsa-let-7f-5p	13067.8	15764.4	9498.2	10322	8570.2	12909.7	8836.5	9914.3	6440.9
hsa-let-7f-5p	13130	15781	9485	10252.3	8594.9	12927.1	8831.6	9904.2	6433.9
hsa-let-7g-5p	805.6	1557.8	565.9	924.6	439.7	653.9	963.4	1163.7	385.8
hsa-let-7i-5p	3495.6	3719.9	3085.6	2339.1	2517.7	2830.2	2926.6	4479.9	2389.8
hsa-miR-100-5p	175	158.7	134	227.2	95.6	122.8	82.4	170.1	74.9
hsa-miR-101-3p	711	578.2	828.8	805.9	356.1	334.7	408.5	582.7	285.8
hsa-miR-101-3p	730.1	598.7	850	855.7	381.9	334.7	432.9	647.2	291.9
hsa-miR-103a-3p	1869.3	3163.6	1375.7	1384.6	1064.3	2850.1	2077.9	2190.6	1407.4
hsa-miR-103a-3p	1869.5	3163.6	1375.7	1394.7	1074.2	2850.1	2077.9	2190.6	1407.4
hsa-miR-106b-3p	37.7	136.3	54.3	22.5	54.1	104.8	70.2	79.4	94.8
hsa-miR-107	307.7	611.9	298.5	242.2	162.4	598.4	314.3	378.4	118.5
hsa-miR-10a-5p	47752.7	30423.9	43826.1	41958.6	43275.7	53358.6	42483.3	33140.9	49844.1
hsa-miR-10b-5p	375699	249858.9	496967.7	577786.3	641228.8	500173.2	588847	509664.1	646598.3
hsa-miR-125a-5p	439.4	440.9	396.2	194.7	134.2	198.2	151.8	78.9	169.3
hsa-miR-125b-2-3p	378.2	380.4	335.2	48.3	99.5	228.1	135	99.5	72.8
hsa-miR-1307-3p	276	624.1	326.2	255.3	211.8	459.7	281.5	249.5	231.4
hsa-miR-141-3p	159	320.3	142.7	71.1	153.8	79.2	239.1	280.6	137.5
hsa-miR-146a-5p	61.2	162.9	108.6	17.6	67.2	82.9	73.2	132.4	25.5
hsa-miR-146b-5p	3154.2	2890.8	2731.8	2357.9	2527	2897.1	1820.5	2167.4	2321.1
hsa-miR-148a-5p	685.3	846	239.7	323.4	313.8	459.1	349.4	888.3	171.4
hsa-miR-148a-3p	6773.7	9253.9	2980.2	2237	1649.4	2286.2	2288.5	3032.9	1268.5
hsa-miR-151a-3p	4205	4274.3	4187.2	2294.6	3323	5087.9	5075.8	3789.7	4475.2
hsa-miR-155-5p	413.5	1258.6	66.2	79	119	68.4	116.7	187.7	66.1
hsa-miR-181a-5p	11000.4	9702.2	15243.3	7930	7362.3	10382.9	10339.6	10286.1	6925
hsa-miR-181a-2-3p	96.7	345.5	157.1	168.5	113.8	162.3	242.2	98.7	48.8
hsa-miR-181a-5p	11000.4	9702.2	15243.3	7930	7362.3	10382.9	10339.6	10286.1	6925
hsa-miR-181b-5p	941.9	1358.4	1280.6	683.1	561.5	1070	810.9	1586.4	431.4
hsa-miR-181b-5p	955.1	1377.5	1280.6	683.1	561.5	1070	820.8	1586.4	431.4
hsa-miR-181c-5p	211.4	86.4	177.4	53.9	324.8	103.7	95.4	241.2	279.9
hsa-miR-181d-5p	107.4	117.6	67.8	116	142.4	26.8	103.7	141.2	114.1
hsa-miR-182-5p	4281.3	5683.8	3110	4836.6	2879.7	4052.3	4799.3	4957.8	3889.7
hsa-miR-183-5p	1176.5	908.1	1558.9	2711.6	679.4	977.2	1234.2	952.8	537.3
hsa-miR-186-5p	365.1	533.1	214.3	181.6	205.6	300.5	206	361.3	108.2
hsa-miR-191-5p	3161.6	3091.2	2381.9	1218.1	1312.8	2396.7	2912.5	1930.2	2174.9
hsa-miR-192-5p	2203.5	2416.6	1868.7	785.7	875	1372.5	1280.8	1639	461.8
hsa-miR-196a-5p	716	609.9	726.3	232.1	421.2	630	466.9	374.5	438.4
hsa-miR-196a-5p	930.2	897.5	886	274.4	557.8	808.9	581.7	502.1	525.6
hsa-miR-200a-5p	65.4	155.3	113.1	74.9	89.1	219.9	111.8	100.9	59.4
hsa-miR-200a-3p	896.5	1171.2	849.3	466.4	669.9	1287.9	1096.6	858.5	841.5
hsa-miR-200b-3p	249.9	457.2	108.9	18.3	88.9	239.5	164	128.9	162.3
hsa-miR-200c-3p	2874.5	4333.2	1541.2	1166.8	1192.9	3960.9	3510.9	2263.4	2159.9

hsa-miR-203a-3p	587.8	780.8	541.8	259.8	250.9	857.3	1204.9	453.8	375.8
hsa-miR-204-5p	3430.8	5399.4	2872.6	2444	3762.2	3440	3259.5	4541.2	2711.3
hsa-miR-205-5p	81.5	296.6	157.8	69.6	12	167.5	175.8	101.7	32.5
hsa-miR-21-5p	3941.3	8137.1	1968.9	1918.8	2101.3	5312.1	5891.6	11676	2060.5
hsa-miR-22-3p	1649.7	1749	1718.6	680.5	597.1	1236.4	963.8	1012	733.5
hsa-miR-221-3p	671.5	968.2	260.6	285.2	462.9	755	459.6	420.1	361.8
hsa-miR-222-3p	668.2	740.3	326.2	564.1	340.9	471.7	379.1	260	496
hsa-miR-25-3p	958.7	1693.9	520.6	698.9	439.9	1436.9	802.1	1424.2	395.4
hsa-miR-26a-5p	24388.7	21129.4	20605	9156.7	12673.1	15471.9	14675.9	12926.1	15032.4
hsa-miR-26a-5p	24388.7	21129.4	20605	9156.7	12673.3	15491.3	14675.9	12926.1	15032.4
hsa-miR-26b-5p	4040.2	6926	2349.7	1070.6	1639.9	2601.2	2195.8	2962.7	1814.8
hsa-miR-27b-3p	299.3	286.6	232.3	140	186.7	225	256.7	155.2	187.5
hsa-miR-28-3p	4400.1	5798.5	3651.5	3108	2131.6	3072.9	3256.5	2598.8	2457.7
hsa-miR-30a-3p	13399.2	16081.5	13298.8	9358.4	15411.2	18678.1	17633.3	26126.2	11802.3
hsa-miR-30a-5p	49940.7	32363.5	58270.5	51403.9	56846	44796.9	48889.9	59147.9	52602.8
hsa-miR-30c-5p	685.5	909.7	555.6	197.3	385.8	621.2	308.9	507.8	451
hsa-miR-30c-2-3p	734.7	530.7	724.7	498.6	174.4	308.7	272.3	476.6	150.6
hsa-miR-30c-5p	685.5	909.7	555.6	197.3	385.8	621.2	308.9	507.8	451
hsa-miR-30d-5p	5135.4	6379.9	4050.3	2957.5	3196.3	4974	3280.9	4073.8	3899.6
hsa-miR-30e-5p	1076.3	932.7	826.5	912.6	677	771	599.6	514.3	740.6
hsa-miR-30e-3p	1674.9	2171.1	1497.5	1879.1	2127	2891.4	2304.5	3183.7	1831.8
hsa-miR-320a	5998	6912.2	3887.4	2226.5	2494.1	4528	2935.7	5534.4	2412.4
hsa-miR-328-3p	190.3	295.2	102.8	70	69.2	141.3	142.3	89.5	138.6
hsa-miR-375	9697.2	15119.7	189.9	1373	962.5	1847.9	2132.5	5413.4	1650.4
hsa-miR-378a-3p	1176.5	1500.3	1370.6	533.4	689.7	1126.2	1127.1	1294.8	677.4
hsa-miR-423-3p	483.6	751.6	438.3	216	229.2	379.7	327.3	395.1	164.7
hsa-miR-423-5p	2237	1889.5	2070.1	1511.1	1234	1824.8	1369.3	1333	1515
hsa-miR-486-5p	1321	768.2	1696.4	2528.9	394.8	424.7	569.1	793.6	195.7
hsa-miR-486-5p	1325.1	774.4	1686.8	2493.7	394.8	424.9	569.1	793.6	196
hsa-miR-532-5p	664	944	523.5	337.6	110.4	110.5	71.7	183.3	103.2
hsa-miR-574-5p	319	596	137.2	109.3	102.7	405.9	174.3	220.6	73.1
hsa-miR-577	144.5	145.3	101.2	141.9	109.1	147.8	185.4	263.5	126.6
hsa-miR-660-5p	104	114.8	134.3	53.2	61.2	146.4	59.5	119.3	109.4
hsa-miR-769-5p	112	142.9	100.9	138.9	125.9	72.9	149.1	222.3	91.3
hsa-miR-9-5p	117.8	168	60.4	391.2	300.9	112.2	166.7	588.9	186.3
hsa-miR-9-5p	117.8	168	60.4	391.2	300.9	112.2	166.7	588.9	186.3
hsa-miR-92a-3p	18245.8	26609.6	14400.4	15437.1	1452.2	3132.7	1865.9	2337.5	1184.5
hsa-miR-92a-3p	18857.4	27672	14700.8	15841.7	1528.9	3290.2	1914.3	2656.3	1267
hsa-miR-92b-3p	555.2	544.7	547.6	174.4	456.2	421	578.6	570.5	633.8
hsa-miR-93-5p	190.7	529.7	170	147.5	65.7	107.7	247.5	197.3	109.1
hsa-miR-9-5p	117.8	168	60.4	391.2	300.9	112.2	166.7	588.9	186.3
hsa-miR-941	190.9	325.7	129.2	181.6	152.3	317.9	85.4	211.8	127.2
hsa-miR-941	190.9	325.7	129.2	181.6	152.3	317.9	85.4	211.8	127.2
hsa-miR-941	190.9	325.7	129.2	181.6	152.3	317.9	85.4	211.8	127.2
hsa-miR-941	190.9	325.7	129.2	181.6	152.3	317.9	85.4	211.8	127.2
hsa-miR-941	190.9	325.7	129.2	181.6	152.3	317.9	85.4	211.8	127.2
hsa-miR-98-5p	979.6	1729	702.5	565.6	551.8	1290.5	916.9	1214.1	448.7
hsa-miR-99a-5p	472.9	396.4	292.4	271.4	209.2	221.3	116	360.9	207.1
hsa-miR-99b-5p	4929.5	2946.3	5716.2	3831.6	3525.3	3780.9	4040.7	2860.6	2756.9
hsa-miR-148b-3p	229.4	366.4	105.1	289.4	56.3	160.9	98.4	288.1	38

hsa-miR-31-5p	69.2	163.1	74.2	6	38.2	105.7	268.5	184.2	92.4
hsa-miR-425-5p	102.8	127.1	96.1	32.9	9.5	60.4	72.1	104.4	72.8
hsa-miR-140-3p	260.1	403.6	325.5	207	68.1	115.6	8.4	163.6	25.2
hsa-miR-151a-5p	61.8	63.9	50.1	0	28.6	164.1	77	139	62
hsa-miR-151b	61.8	63.9	50.1	0	28.6	164.1	77	139	62
hsa-miR-152-3p	83.3	79.8	68.5	46.8	40	40.2	21.4	79.4	88.6
hsa-miR-23b-3p	35.5	127.9	27.3	20.2	35.2	132.7	67.1	230.2	32.2
hsa-miR-24-3p	71.9	69.1	81.3	0	23.2	17.1	42.7	69.3	33.3
hsa-miR-24-3p	71.9	69.1	81.3	0	23.4	17.1	42.7	69.3	33.3
hsa-miR-421	23.3	79.6	28.6	21	67	31.3	101.5	142.5	41.8
hsa-miR-744-5p	41.7	83.8	73.9	24.3	19.6	71.8	99.6	46.9	29.3
hsa-miR-16-5p	31.9	42.5	62.7	1.1	14.6	56.1	1.9	0.9	103
hsa-miR-16-5p	31.9	42.5	62.7	1.1	14.6	56.1	1.9	0.9	103
hsa-miR-196b-5p	244.9	359.4	178.4	60.6	56.9	48.7	0	56.1	36
hsa-miR-363-3p	140.5	206.8	46.9	46.4	41.7	91.7	24.4	94.3	60.8
hsa-miR-574-3p	105.8	259.1	54	0	47.5	259.2	140.7	46	19.3
hsa-miR-891a-5p	153.2	311.3	0.3	422.6	230.9	243	32.8	355.2	208
hsa-miR-99b-3p	65.6	71.6	53.3	31.4	18.7	157.5	53	92.5	34.2
hsa-miR-1180-3p	103.2	68.3	108.3	0	30.9	78.6	64.1	68.8	52.9
hsa-miR-30b-5p	40.3	63.5	31.8	6.7	32.4	108.8	45.4	42.5	23.4
hsa-miR-30b-3p	17.3	43.7	16.7	12.4	34.6	53.8	70.2	97.3	59.7
hsa-miR-4446-3p	53.4	41.1	15.8	17.6	21.5	25.1	111.8	103.9	22.2
hsa-miR-340-5p	47.4	60.3	16.1	15	14.6	38.7	21.4	26.8	0
hsa-miR-598-3p	66.4	77.2	39.2	27.3	15	69.5	29.4	38.6	0
hsa-miR-210-3p	61.2	55.5	41.8	16.1	8.4	74.1	0	20.2	11.7
hsa-miR-28-5p	51	59.9	8.4	14.2	19.8	80.6	98.8	15.8	14
hsa-miR-3615	25.3	53.9	58.8	41.2	0	29.6	70.6	92.5	31.3
hsa-miR-374a-3p	33.5	21	10.6	11.6	50.3	0	63.3	33.3	36.6
hsa-miR-425-3p	18.3	47.5	28	0	44.5	32.2	17.2	27.2	13.2
hsa-miR-7706	7.6	32.1	49.2	7.9	44.5	17.9	0	36	32.5
hsa-miR-130a-3p	56.2	49.7	103.2	0	83.6	13.1	33.2	0	36.9
hsa-miR-184	152	62.3	168.4	110.4	31.1	52.4	16.8	56.6	0
hsa-miR-19b-3p	27.5	72.2	7.4	34.1	31.4	36.2	29.4	25	22.2
hsa-miR-19b-3p	27.5	72.2	7.4	34.1	31.4	36.2	29.4	25	22.2
hsa-miR-429	35.3	141.5	26.7	32.9	9	37	19.8	133.3	14
hsa-miR-92b-5p	15.3	25.1	18	0	23.2	20.2	0	20.2	11.4
hsa-miR-342-3p	39.1	52.1	64.3	18	45.3	22.8	0	0	25.5
hsa-miR-500a-3p	138.9	204	207	102.2	15	10.8	16	0	0
hsa-miR-181a-3p	14.8	37.5	30.9	21.3	9.2	15.7	0	0	12.3
hsa-miR-320b	28.1	43.9	30.5	35.9	30.1	13.4	38.1	28.9	2.3
hsa-miR-361-5p	30.9	50.9	18	19.1	20.6	16.2	0	75.4	40.4
hsa-miR-424-3p	28.3	37.5	66.2	10.9	45.1	71.5	38.9	81.6	0
hsa-miR-92a-1-5p	45.4	73.8	18.3	37.8	27.7	81.7	0	44.7	34.2
hsa-miR-320b	28.1	62.3	30.5	35.9	30.1	3.4	37.8	28.5	2.3
hsa-miR-345-5p	69.2	83.6	26	0	24.1	28.2	2.7	0	0
hsa-miR-584-5p	25.9	110.6	53.7	16.8	4.9	41.6	117.9	32	0
hsa-miR-671-3p	55.4	45.9	0	0	0	17.4	38.9	21.1	5.6
hsa-miR-1226-5p	22.5	33.7	10.6	71.9	12.5	0	19.8	0	0
hsa-miR-1270	16.5	17.6	0	0	17.2	38.7	0	28.9	0
hsa-miR-218-5p	10.6	13.8	6.4	44.2	12.7	38.2	0	20.2	9.1
hsa-miR-218-5p	10.6	13.8	6.4	44.2	12.7	38.2	0	20.2	9.1

hsa-miR-29a-3p	15.1	34.9	8.4	0	12	39.9	24	31.1	0
hsa-miR-342-5p	41.9	64.9	51.4	32.2	0	49.8	0	0	30.1
hsa-miR-1468-5p	23.9	17.2	11.9	16.8	0	0	0	0	0
hsa-miR-29c-3p	22.1	23.5	11.9	18.3	2.2	28.8	22.1	11	24.6
hsa-miR-324-5p	19.3	28.9	0	0	0	23.4	0	0	0
hsa-miR-365b-5p	16.9	27.3	25.4	15.4	10.7	30.2	29.4	18	0
hsa-miR-432-5p	9	101.4	100.3	0	0	72.6	42.3	63.1	10.8
hsa-miR-99a-3p	19.9	26.9	27	0	0	8.5	28.6	21.5	0
hsa-miR-10b-3p	32.3	8.2	19	40.8	18.3	19.9	18.3	0	58.8
hsa-miR-194-5p	49.4	33.5	29.6	0	0	20.8	0	57.4	24.9
hsa-miR-197-3p	15.3	36.3	14.5	0	6.4	17.4	18.3	14	9.4
hsa-miR-132-3p	23.9	58.5	17	17.6	0	0	0	25.9	0
hsa-miR-146b-3p	7.6	10	0	0	0	33.3	20.6	0	0
hsa-miR-194-5p	49.2	33.3	28.9	0	0	20.8	0	57.4	24.9
hsa-miR-365a-5p	19.1	14.6	0	18.7	20.2	0	19.5	69.7	0
hsa-miR-484	27.5	28.9	15.1	0	15.5	17.7	0	37.3	9.9
hsa-miR-128-3p	6.6	5.8	11.6	13.1	27.1	13.4	0	68.4	5.6
hsa-miR-27b-5p	21.1	17.2	0	0	24.5	0	0	0	0
hsa-miR-589-5p	13.8	25.5	9	20.2	0	0	6.1	19.7	0
hsa-miR-125b-5p	9.6	10.6	6.8	0	17.2	0.3	0	40.3	9.4
hsa-miR-125b-5p	9.6	10.6	6.8	0	17.2	0.3	0	40.3	9.4
hsa-miR-128-3p	6.6	5.8	11.6	0	40	13.4	0	68.4	0
hsa-miR-181c-3p	0.2	5	0	18	14	20.5	0	0	0
hsa-miR-30d-3p	24.7	37.5	24.4	3.7	3.7	23.1	3.8	7.5	2.9
hsa-miR-361-3p	28.5	44.1	15.4	16.8	0	0	19.8	37.3	14.6
hsa-miR-501-3p	35.3	11	46.6	110.8	10.1	0	0	25	0
hsa-miR-874-3p	55.2	38.5	8.7	15	5.2	17.4	0	0	0
hsa-miR-130b-3p	7.4	30.5	22.5	15	0	20.2	0	0	0
hsa-miR-200b-5p	13.8	10.8	7.7	14.6	0	0	0	0	13.2
hsa-miR-204-3p	24.1	20	5.5	16.5	8.2	0.6	0	0	19.3
hsa-miR-409-3p	0	22.5	27.3	71.9	18.3	35	30.9	0	12.9
hsa-miR-7-5p	12	36.1	20.9	19.1	0	5.1	0	84.6	0
hsa-miR-7-5p	12	36.1	20.9	19.1	0	5.1	0	84.6	0
hsa-miR-7-5p	15	36.1	20.9	19.1	0	5.1	0	84.6	0
hsa-miR-873-3p	24.1	9	40.5	16.1	26	0	0	0	21.9
hsa-let-7a-3p	10.6	31.5	18.3	0	0	0	21.7	23.2	0
hsa-let-7a-3p	10.6	31.5	18.3	0	0	0	21.7	23.2	0
hsa-miR-1262	12.8	11.2	15.8	7.5	0	0	15.6	18	0
hsa-miR-1269a	3.6	0	33.7	15.7	0	0	0	0	0
hsa-miR-143-3p	8.2	0.6	34.1	83.5	29	2.3	0	89.9	52.9
hsa-miR-3622a-5p	0	4.2	16.4	0	9.9	0	0	0	14.6
hsa-miR-449c-5p	20.9	31.1	38.2	0	0	0	0	0	0
hsa-miR-92a-2-5p	43.2	36.3	0	0	0	0	16.4	0	18.1
hsa-miR-1307-5p	7.6	6.4	0	41.2	0	17.7	0	0	0
hsa-miR-17-5p	11.8	17.4	19.3	0	0	0	0	0	0
hsa-miR-20b-5p	15.3	14.4	0	0	0	0	0	0	0
hsa-miR-30c-1-3p	9.4	11.2	18	0.8	15.9	0.3	0.4	0	9.4
hsa-miR-542-3p	17.7	0	24.1	0	24.1	0	0	0	0
hsa-miR-642a-5p	5.4	4.8	12.9	19.8	0	0	0	3.5	18.7

hsa-miR-6859-5p	13.6	3.2	0	31.4	16.3	0	0	0	0
hsa-miR-6859-5p	13.6	3.2	0	31.4	16.3	0	0	0	0
hsa-miR-6859-5p	13.6	3.2	0	31.4	16.3	0	0	0	0
hsa-miR-6859-5p	13.6	3.2	0	31.4	16.3	0	0	0	0
hsa-miR-877-5p	11.2	24.9	26.7	35.6	0	0	0	14.9	10.2
hsa-miR-887-3p	42.2	19	34.1	0	0	48.4	0	0	0
hsa-miR-128-1-5p	7.2	17.8	0	0	0	0	0	0	0
hsa-miR-3158-3p	0	7.6	9.3	19.1	7.1	0	0	0	0
hsa-miR-3158-3p	0	7.6	9.3	19.1	7.1	0	0	0	0
hsa-miR-3605-5p	10.8	5	20.9	0	0	0	17.2	0	0
hsa-miR-382-5p	0	25.7	8.4	38.2	0	0	0	0	0
hsa-miR-4728-3p	4.6	15.6	0	0	8.2	16.8	21.4	24.1	9.1
hsa-miR-629-5p	14	10.2	12.5	28.5	3.7	0	0	0	0
hsa-miR-6863	15.7	4.4	9.3	0	9	0	0	26.3	0
hsa-miR-760	9.4	11.4	0	11.2	8.4	0	15.3	35.5	0
hsa-let-7b-3p	5	20	0	0	12.5	0	19.5	30.3	0
hsa-miR-100-3p	0	13.4	12.2	14.6	0	0	0	28.1	10.8
hsa-miR-127-3p	5	18.2	30.9	20.2	1.7	4.8	0	20.6	2.1
hsa-miR-151b	8.6	10	9	0	6	14.8	27.1	18	0
hsa-miR-194-3p	2.2	24.5	20.6	0	0	16.8	0	28.5	0
hsa-miR-20a-5p	4.8	6.4	0	0	0	0	0	53.1	0
hsa-miR-2110	0	19.6	18	0	0	8.8	0	0	9.7
hsa-miR-489-3p	0	6.2	0	0	0	0	16.4	0	0
hsa-miR-509-3p	32.3	11.6	27	0	18.7	0	0	0	0
hsa-miR-509-3p	32.3	11.6	27	0	18.7	0	0	0	0
hsa-miR-509-3p	32.3	11.6	27	0	18.7	0	0	0	0
hsa-miR-548az-5p	0	0.6	28.3	18	8.6	0	0.4	25	8.8
hsa-miR-10a-3p	12.6	11.2	0	0	0	16.5	0	0	0
hsa-miR-125b-1-3p	9.4	6.8	17	9.7	6.7	0	38.1	0.9	7
hsa-miR-1269b	0	0	0	0	10.1	0	0	0	0
hsa-miR-1304-3p	4.4	9.4	22.5	30.3	0	16	0	0	0
hsa-miR-193b-5p	22.3	9.4	0	15.7	0	0	0	14.9	0
hsa-miR-195-3p	13	11.2	0	0.8	0	0	0	0	0
hsa-miR-23a-3p	10.8	2.6	0	0	0.2	0	0.4	49.1	14.3
hsa-miR-29b-3p	12.8	10.8	10.9	0	0	0	0	0	0
hsa-miR-29b-3p	12.8	10.8	10.9	0	0	0	0	0	0
hsa-miR-29b-2-5p	5.4	0	0	0	0	15.4	0	0	12.6
hsa-miR-301a-3p	5.2	1.8	7.4	14.2	4.1	0	0	0	0
hsa-miR-335-3p	9.4	12.8	16.7	0	0.2	0	0	0	0.9
hsa-miR-34a-5p	10.6	16.2	8.4	0	5.6	19.7	22.1	0	0
hsa-miR-3605-3p	0	11.6	0	8.6	0.2	0	0	0	0
hsa-miR-452-5p	0	4.8	11.3	0	0	0	18.3	0	0
hsa-miR-455-5p	0	8.2	0	0	0	32.8	0	0	0
hsa-miR-664a-5p	8.8	6.8	11.6	0	0	0	21	0	0
hsa-miR-708-5p	0	9.8	0	21.3	0	0	22.9	10.1	0
hsa-miR-769-3p	0	9	7.4	0	0	17.1	0	0	22.8
hsa-miR-892a	0	11.6	0	0	17.2	16.8	0	0	0
hsa-miR-106a-5p	4	15.8	0	0	0	0	0	0	12.9
hsa-miR-148b-5p	0	6.8	0	0	0	3.1	0	29.4	0
hsa-miR-193b-3p	9.8	6.4	15.8	0	0	0	0	0	11.4
hsa-miR-3065-3p	10	10	4.2	0	0	0	0	0	11.7
hsa-miR-330-5p	0	0	0	0	0	17.1	0	0	0
hsa-miR-582-3p	0	10.4	9.3	37.4	9.2	0	0	0	0
hsa-miR-625-3p	0	5.4	14.8	0	0	17.9	0	0	9.7
hsa-miR-939-5p	22.3	4	0	0	0	0	28.2	0	0

S7 Table
